# Classification of Drugs Based on Properties of Sodium Channel Inhibition: A Comparative Automated Patch-Clamp Study

**DOI:** 10.1371/journal.pone.0015568

**Published:** 2010-12-20

**Authors:** Nora Lenkey, Robert Karoly, Peter Lukacs, E. Sylvester Vizi, Morten Sunesen, Laszlo Fodor, Arpad Mike

**Affiliations:** 1 Department of Pharmacology, Institute of Experimental Medicine, Hungarian Academy of Sciences, Budapest, Hungary; 2 Sophion Bioscience A/S, Ballerup, Denmark; 3 Pharmacology and Drug Safety Research, Gedeon Richter Plc., Budapest, Hungary; Biological Research Center of the Hungarian Academy of Sciences, Hungary

## Abstract

**Background:**

There is only one established drug binding site on sodium channels. However, drug binding of sodium channels shows extreme promiscuity: ∼25% of investigated drugs have been found to potently inhibit sodium channels. The structural diversity of these molecules suggests that they may not share the binding site, and/or the mode of action. Our goal was to attempt classification of sodium channel inhibitors by measuring multiple properties of inhibition in electrophysiology experiments. We also aimed to investigate if different properties of inhibition correlate with specific chemical properties of the compounds.

**Methodology/Principal Findings:**

A comparative electrophysiological study of 35 compounds, including classic sodium channel inhibitors (anticonvulsants, antiarrhythmics and local anesthetics), as well as antidepressants, antipsychotics and neuroprotective agents, was carried out using rNav1.2 expressing HEK-293 cells and the QPatch automatic patch-clamp instrument. In the multi-dimensional space defined by the eight properties of inhibition (resting and inactivated affinity, potency, reversibility, time constants of onset and offset, use-dependence and state-dependence), at least three distinct types of inhibition could be identified; these probably reflect distinct modes of action. The compounds were clustered similarly in the multi-dimensional space defined by relevant chemical properties, including measures of lipophilicity, aromaticity, molecular size, polarity and electric charge. Drugs of the same therapeutic indication typically belonged to the same type. We identified chemical properties, which were important in determining specific properties of inhibition. State-dependence correlated with lipophilicity, the ratio of the neutral form of molecules, and aromaticity: We noticed that the highly state dependent inhibitors had at least two aromatic rings, logP>4.0, and pKa<8.0.

**Conclusions/Significance:**

The correlations of inhibition properties both with chemical properties and therapeutic profiles would not have been evident through the sole determination of IC_50_; therefore, recording multiple properties of inhibition may allow improved prediction of therapeutic usefulness.

## Introduction

Pharmacological modulation of sodium channels by sodium channel inhibitors (SCIs) is crucial in local anesthesia, in the treatment of certain types of epilepsy and cardiac arrhythmia (we will refer to these drugs: local anesthetics, anticonvulsants and class I antiarrhythmics, as classic SCIs). Several SCI drugs are also used for the treatment of neuropathic pain, muscle spasms, Alzheimer's disease, amyotrophic lateral sclerosis and as mood stabilizers [1], although in some of these indications the role of sodium channel inhibition is unsettled. Furthermore, SCIs are intensively studied (preclinical/clinical phase) for a number of other indications including various pain syndromes, stroke/ischemia, neurodegenerative diseases (Parkinson's disease, multiple sclerosis), and psychiatric disorders [1], [2]. The basis of the therapeutic versatility of SCIs is poorly understood. Isoform selectivity, which would be the most plausible explanation, is minimal for most SCIs [3]. Instead, it is conceivable that different therapeutic profiles are caused by different mechanisms of action, such as different binding sites, different access pathways to the binding site [4] and different state-selectivity [5]. Current knowledge regarding the relationship between chemical properties of SCIs, biophysical properties of inhibition (reflecting mechanism of action) and therapeutic profile is limited.

There are several different toxin binding sites on sodium channels [6], [7], but only one established drug binding site. Therefore, it was puzzling to learn that according to a recent study, about 25% of clinically used drugs were sodium channel inhibitors [8]. The criteria for being classified as a sodium channel inhibitor in that study was to cause at least 60% inhibition at 10 µM concentration; notably, classic SCIs such as lidocaine or lamotrigine failed to fulfill this criteria. A “local anesthetic receptor”, which can host every fourth drug with an affinity higher than that of lidocaine, is at least curious. Furthermore, the identity of residues involved in drug binding seems to vary from drug to drug [9]. Even the contribution of the best established component of the binding site, Phe1764 (rNav1.2 numbering), was found to be minimal in 8 out of the 28 SCI compounds studied thus far [9]. The overall picture suggests multiple overlapping binding sites within the inner vestibule, and for certain drugs completely different binding sites. Furthermore, we also know that binding sites at different conformations of the channel are fundamentally different, and most probably impose different orientations and positions on bound drugs [10], [11], [12]. We assumed that binding to different binding sites (either within the supposed overlapping multi-binding site of the inner vestibule, or out-of-inner-vestibule binding sites), or preferring different conformation of the binding site should be reflected in the experimentally measurable properties of inhibition. We aimed to measure properties of inhibition in order to obtain indirect information regarding possible binding sites and/or modes of action. For this purpose, it is essential to acquire more information than simply determining the concentration-response relationship.

The way the acquisition of multiple biophysical properties was carried out was unlike conventional analyses of the mode of action. These require multiple protocols, where the exact parameters of protocols must be adjusted individually to specific drugs. Furthermore, exploration of the mode of action of a drug is always an iterative process: responses to initial protocols are used to design new protocols, and to adjust parameters of the protocols to individual drugs. This of course compromises comparability of data with different drugs. Our aim is to develop an approach which can be adopted by pharmaceutical companies, therefore we confined ourselves to protocols which require no more cost or time than a single measurement of inhibition, but which can give radically more information regarding the mode of action of the drug. We used two very simple protocols, which can even be applied within the same experiment, the whole process of ‘control measurement’ – ‘measurement of drug effect’ – ‘measurement after washout’ requires no more than 10 minutes (it is limited by the onset rate of slowly acting drugs). However, we took care to extract all possible useful information from this measurement.

From the first protocol, where 5 Hz trains of depolarizations were applied, we extracted potency, reversibility, time constants of onset and offset and use-dependence. Affinity to hyperpolarized state (commonly termed “resting state affinity”; K_r_), affinity to depolarized states (commonly termed “inactivated state affinity”; K_i_), and state dependence (calculated from the ratio of K_r_ and K_i_) were extracted from “steady-state” availability curves (for details of the protocol, see Materials and Methods).

We investigated 44 compounds of different chemical structures and different therapeutic classes (Table 1). Twelve of the compounds (nisoxetine, mirtazapine, bupropion, nefazodone, nialamide, moclobemide, chlorprothixene, clozapine, tiapride, ritanserin, mecamylamine, deprenyl), to the best of our knowledge, have not been tested before for SCI activity. Most drugs were acting on the CNS, we placed special emphasis on antidepressants, which, intriguingly, were found to be the therapeutic group with the highest incidence (72%) of SCI activity in the above quoted study of ∼400 drugs [8]. Nine of the 44 compounds did not produce effective inhibition even at the highest concentrations used. For the remaining 35 drugs, the comparative electrophysiological investigation was completed. Recording multiple biophysical properties for these drugs made it possible to delineate distinct groups in the multi-dimensional “biophysical space” (defined by biophysical properties of inhibition; in analogy to the “chemical space” defined by chemical descriptors [13]), which correspond to distinct types of inhibition. The identified types correlated significantly with therapeutic categories.

**Table 1:** List of drugs with three-letter codes, therapeutic indications, main protein targets and plasma concentrations of sodium channel inhibitor.

Drug	Code	Main use	Mechanism of action	plasma conc.	
				µg/ml	µM
Fluoxetine	FLX	antidepressant	SSRI a	0.38 [44]	1.099
Sertraline	SRT	antidepressant	SSRI a	0.14 [44]	0.409
Paroxetine	PRX	antidepressant	SSRI a	0.38 [44]	0.22
Amitriptyline	AMI	antidepressant	TCA b	0.064 [44]	0.204
Imipramine	IMI	antidepressant	TCA b	0.2 [44]	0.631
Desipramine	DMI	antidepressant	TCA b	0.1 [44]	0.330
Maprotiline	MPR	antidepressant	NRI c	0.25 [45]	0.797
Nisoxetine	NIS	antidepressant	NRI c	0.05 [46]	0.159
Mianserin	MIA	antidepressant	NaSSA d	0.01 [47]	0.037
Mirtazapine	MRZ	antidepressant	NaSSA d	0.04 [44]	0.158
Bupropion	BPR	antidepressant	NDRI e (Augmenter)	0.14 [44]	0.514
Venlafaxine	VFX	antidepressant	SNRI f	0.17 [44]	0.532
Nefazodone	NFZ	antidepressant	SNRI f (Augmenter)	1.52 [48]	3.007
Trazodone	TRZ	antidepressant	(Augmenter)	1.5 [44]	3.673
Nialamide	NIA	antidepressant	non-sel.MAO^g^ inh.	NDA	NDA
Moclobemide	MCL	antidepressant	MAO g -A inhib.	0.29 [49]	1.484
Haloperidol	HAL	antipsychotic	D_2_ antagonist	0.02 [44]	0.053
Chlorpromazine	CPM	antipsychotic	D_2_ antagonist	0.1 [44]	0.281
Chlorprothixene	CHX	antipsychotic	D_2_ antagonist	0.07 [50]	0.200
Clozapine	CLZ	antipsychotic	D_4_ antagonist	0.55 [44]	1.671
Tiapride	TIA	antipsychotic	D_2_ antagonist	0.56 [51]	1.535
Carbamazepine	CBZ	antiepileptic	SCI h	9.3 [44]	39.362
Lamotrigine	LTG	antiepileptic	SCI h	2.5 [44]	9.762
Phenytoin	DPH	antiepileptic, 1b antiarrhythmic	SCI h	10 [44]	39.64
Topiramate	TOP	antiepileptic	SCI h	5.5 [44]	16.21
Zonisamide	ZON	antiepileptic	SCI h	28.4 [44]	133.82
Gabapentin	GAB	antiepileptic	Ca^2+^-ch. inhib.	4 [44]	23.359
Bupivacaine	BPV	local anesthetic	SCI h	0.8 [44]	2.462
Lidocaine	LID	local anesthetic, 1b antiarrhythmic	SCI h	3.5 [44]	12.925
Mexiletine	MEX	1b antiarrhythmic	SCI h	2 [44]	9.271
Flecainide	FLC	1c antiarrhythmic	SCI h	0.46 [44]	0.965
Procainamide	PRC	1a antiarrhythmic	SCI h	2.5 [44]	9.198
Ranolazine	RAN	antianginal	SCI h	3.59 [52]	7.173
Memantine	MEM	Alzheimer's - dis.	NMDA antagonist	0.02 [53]	0.075
Riluzole	RIL	Amyotrophic lateral sclerosis	SCI h, glutamate release inhibitor	0.17 [44]	0.739
Diclofenac	DIC	NSAID i	COX^J^ inhibitor	2 [44]	6.287
Ritanserin	RIT	anxiolytic	5HT_2A_-_C_ antag.	0.14 [54]	0.299
Ambroxol	AMB	analgesic, mucolytic	SCI h	0.15 [55]	0.362
Silperisone	SIL	muscle relaxant	SCI h	0.45 [56]	1.484
Tolperisone	TOL	muscle relaxant	SCI h	0.43 [57]	1.508
Flunarizine	FLR	migraine	Ca^2+^-ch. inhib.	0.06 [58]	0.113
Lifarizine	LIF	neuroprotective	SCI h, Ca^2+^-ch. inh.	0.02 [59]	0.046
Mecamylamine	MEC	smoking cessation	nACh antagonist	0.02 [60]	0.118
Deprenyl	DPR	Parkinson's – dis.	MAO g -B inhib.	0.001[44]	0.005

Abbr.:

a selective serotonin reuptake inhibitor,

b tricyclic antidepressant,

c norepinephrine reuptake inhibitor,

d noradrenergic and specific serotonergic antidepressant,

e norepinephrine-dopamine reuptake inhibitor,

f serotonin-norepinephrine reuptake inhibitor,

g monoamino-oxidase,

h sodium channel inhibitor,

i non-steroidal anti-inflammatory drug,

j cyclooxigenase-inhibitor.

Next, we aimed to investigate how specific locations in the “biophysical space” correspond to specific chemical properties, i.e., locations in “chemical space”.

Some potentially useful general principles have already been discovered. Lipophilicity, pKa (acidic dissociation constant), and the size of the molecule were proposed to be the most important predictors of properties of inhibition [14].

Lipophilicity can be quantified by either logP, which is the logarithm of the octanol-water partition coefficient of the neutral form of the molecule, or logD (logarithm of the distribution coefficient), which measures the distribution of all forms that are present at a certain pH value. Because the pKa of the studied SCIs are typically between 7.0 and 10.7, most drugs are predominantly positively charged at neutral pH, and lose their charge at higher pH values. For this reason, logD is increasing with higher pH, approaching logP as an upper limit. A linear correlation was found between logP and the logarithm of IC_50_ values [14], [15], [16], [17], [18].

Because local anesthetics are predominantly charged, basic compounds, a pKa value between 7.5 and 10 was considered a requirement for being an effective SCI. However, SCI anticonvulsants are predominantly neutral, and several very potent neutral SCIs has been discovered recently [19]. In a recent comparative analysis of 139 compounds from 73 publications we found no correlation between pKa and potency [19]. The pKa value nevertheless is a major determinant of which access pathways to the binding site are passable for the drug, and thus has been shown to affect onset/offset kinetics and use-dependence [4], [20], [21].

Molecular weight has been shown to correlate with potency [17], and with the kinetics of inhibition [22], [23]. More exactly it was not molecular weight that best predicted recovery kinetics, but the width of the molecule at the aromatic end [24].

Most of these results were based on studies of a single chemical and therapeutic class of SCIs. In this current study we attempted to detect similar general principles for a larger, more diverse group of SCIs. Our specific questions were the following:

- Which measure of lipophilicity, logP or logD better predicts potency?
- Are there chemical descriptors which specifically predict individual biophysical properties of inhibition? Most importantly, can we find some difference between descriptors which determine K_r_ and with K_i_? If yes, then we can deduce something regarding the chemical interactions of the drug with resting and inactivated conformations of the channel. High state-dependence is considered to be essential for good therapeutical applicability. Which chemical properties predict high state-dependence?
- Which chemical properties determine specific types of inhibition (i.e., can we predict the type of inhibition from the chemical structure)?

We succeeded to identify chemical descriptors which determine different types of inhibition, and which predict specific biophysical properties of inhibition (K_r_, K_i_, IC_50_, state-dependence, reversibility, use-dependence, onset and offset time constants). We observed that different chemical properties determine low K_r_ (high pKa and logP), and low K_i_ (high logP, logD and aromaticity). State-dependence, therefore can be predicted by calculating these chemical descriptors of molecules.

## Results

### Assessment of properties of inhibition

Properties of drugs were tested using two simple protocols.

The dynamics of the onset and offset of inhibition was monitored using 5 Hz trains of five depolarizing pulses from a holding potential of −90 mV to −10 mV. The trains were repeated every 20 s. Drugs were applied after 10 control trains and 10 trains were delivered in the presence of the drug. The drug was then washed out, and the 10 trains were repeated. Each drug was applied at a single concentration, which was chosen to cause 25% to 75% inhibition (pilot experiments established appropriate concentrations). Of the 44 investigated drugs nine either caused less than 10% inhibition even at the highest concentration used (mecamylamine, tiapride, topiramate, zonisamide, procainamide, gabapentin, moclobemid, nialamide), or their IC_50_ were exceedingly high compared to their plasma concentration (mecamylamine, deprenyl). Therefore these were excluded from analysis of further properties of inhibition (marked “dropout” in Table 2).

**Table 2:** Values of five biophysical properties for individual drugs extracted from the 5 Hz train protocol.

Drug	cc a	n	Inh b	±	SEM	IC50	Rev c	±	SEM	UD d	±	SEM	τon	±	SEM	τoff	±	SEM
code	μM					μM							s			s		
**A N T I D E P R E S S A N T S**																		
**FLX**	**30**	5	0.41	**±**	0.08	43.09	0.33	**±**	0.09	1.14	**±**	0.01	53.38	**±**	4.86	39.34	**±**	12.4
**SRT**	**30**	7	0.68	**±**	0.07	14.31	0.23	**±**	0.02	1.42	**±**	0.03	26.55	**±**	7.93	29.55	**±**	10.5
**PRX**	**30**	6	0.65	**±**	0.06	16.23	0.34	**±**	0.08	1.46	**±**	0.04	32.07	**±**	5.25	35.99	**±**	5.86
**AMI**	**30**	7	0.56	**±**	0.07	23.46	0.50	**±**	0.09	1.66	**±**	0.05	10.54	**±**	1.42	16.95	**±**	3.84
**IMI**	**30**	5	0.61	**±**	0.04	19.46	0.43	**±**	0.05	1.51	**±**	0.05	16.97	**±**	2.24	16.03	**±**	2.26
**DMI**	**30**	5	0.62	**±**	0.03	18.28	0.43	**±**	0.06	1.32	**±**	0.06	32.02	**±**	3.93	51.82	**±**	4.99
**MPR**	**10**	5	0.30	**±**	0.04	23.03	0.34	**±**	0.07	1.17	**±**	0.04	22.07	**±**	2.39	25.21	**±**	1.1
**NIS**	**100**	6	0.57	**±**	0.06	75.72	0.66	**±**	0.05	1.33	**±**	0.04	20.71	**±**	2.25	23.41	**±**	3
**MIA**	**30**	5	0.41	**±**	0.06	42.77	0.7	**±**	0.13	1.25	**±**	0.04	17.78	**±**	3.35	16.98	**±**	3.35
**MRZ**	**100**	7	0.43	**±**	0.08	132.28	0.89	**±**	0.03	1.14	**±**	0.01	3.99	**±**	0.01	7.47	**±**	1.05
**BPR**	**100**	4	0.15	**±**	0.04	565.02	0.92	**±**	0.03	1.04	**±**	0.02	4.6	**±**	1.16	12.45	**±**	2.77
**VFX**	**100**	4	0.12	**±**	0.02	726.99	0.89	**±**	0.06	1.05	**±**	0.01	4.85	**±**	2.04	5.2	**±**	1.63
**NFZ**	**30**	4	0.39	**±**	0.09	46.43	0.51	**±**	0.09	1.22	**±**	0.04	8.75	**±**	0.57	10.93	**±**	0.63
**TRZ**	**100**	5	0.16	**±**	0.04	365.11	0.67	**±**	0.10	1.02	**±**	0.01	8.25	**±**	2.42	16.64	**±**	3.02
**A N T I P S Y C H O T I C S**																		
**HAL**	**30**	4	0.59	**±**	0.09	20.44	0.59	**±**	0.02	1.56	**±**	0.07	11.91	**±**	2.75	24.11	**±**	5.9
**CPM**	**30**	4	0.41	**±**	0.08	47.28	0.07	**±**	0.03	1.30	**±**	0.06	14.81	**±**	2.44	9.67	**±**	2.18
**CHX**	**30**	4	0.28	**±**	0.05	42.54	0.18	**±**	0.05	1.25	**±**	0.07	7.24	**±**	3.85	14.41	**±**	1.11
**CLZ**	**100**	5	0.51	**±**	0.03	94.54	0.63	**±**	0.02	1.31	**±**	0.02	10.38	**±**	0.51	16.4	**±**	2.71
**A N T I C O N V U L S A N T S**																		
**CBZ**	**300**	5	0.29	**±**	0.02	718	0.87	**±**	0.05	0.98	**±**	0.02	3	**±**	-	10.01	**±**	2.33
**LTG**	**300**	4	0.25	**±**	0.03	904.86	0.85	**±**	0.09	0.97	**±**	0.01	8.19	**±**	1.23	18.1	**±**	1.32
**DPH**	**300**	6	0.18	**±**	0.03	1392.4	0.75	**±**	0.07	1.01	**±**	0.01	5.20	**±**	1.20	15.96	**±**	3.86
**L O C A L A N E S T H E T I C S / A N T I A R R H Y T H M I C S**																		
**BPV**	**100**	4	0.60	**±**	0.04	67.72	0.75	**±**	0.05	1.62	**±**	0.05	7.95	**±**	0.05	16.73	**±**	1.43
**LID**	**300**	6	0.23	**±**	0.02	1084.4	0.95	**±**	0.06	1.14	**±**	0.03	3	**±**	-	6	**±**	0.31
**MEX**	**300**	4	0.57	**±**	0.04	230.54	0.78	**±**	0.04	1.39	**±**	0.03	8.6	**±**	0.87	12.93	**±**	0.72
**FLC**	**300**	4	0.82	**±**	0.03	67.63	0.57	**±**	0.04	1.34	**±**	0.02	20.04	**±**	1.26	64.97	**±**	5.51
**M I S C E L L A N E O U S**																		
**RAN**	**100**	4	0.16	**±**	0.04	354.55	0.89	**±**	0.07	1.21	**±**	0.02	8.13	**±**	1.75	13.24	**±**	1.98
**MEM**	**100**	5	0.32	**±**	0.02	230.49	0.93	**±**	0.05	0.99	**±**	0.03	3.51	**±**	0.22	8.98	**±**	1.61
**RIL**	**30**	4	0.27	**±**	0.07	80.98	0.52	**±**	0.08	0.95	**±**	0.03	3.9	**±**	0.07	17.6	**±**	5.42
**DIC**	**100**	6	0.56	**±**	0.1	561.19	0.95	**±**	0.04	1.01	**±**	0.04	7.19	**±**	1.89	26.43	**±**	5.31
**RIT**	**30**	5	0.15	**±**	0.06	23.62	0.37	**±**	0.02	1.09	**±**	0.00	14.38	**±**	2.64	22.28	**±**	6.47
**AMB**	**100**	5	0.51	**±**	0.1	95.94	0.81	**±**	0.06	1.24	**±**	0.02	5.74	**±**	1.14	6.78	**±**	1.1
**SIL**	**100**	6	0.45	**±**	0.06	120.65	0.8	**±**	0.04	1.16	**±**	0.02	4.1	**±**	0.56	15.21	**±**	4.12
**TOL**	**100**	5	0.23	**±**	0.01	335.95	0.95	**±**	0.03	1.14	**±**	0.04	5.57	**±**	1.57	7.42	**±**	1.47
**FLR**	**100**	4	0.85	**±**	0.04	17.65	0.004	**±**	0.001	1.06	**±**	0.03	132.8	**±**	23.5	500	**±**	-
**LIF**	**30**	4	0.78	**±**	0.06	5.29	0.01	**±**	0.001	1.02	**±**	0.03	96.5	**±**	36.8	500	**±**	-
**D R O P O U T** e																		
**MEC**	**100**	4	0.05	**±**	0.01	2757.1												
**TIA**	**300**	4	0.07	**±**	0.02	3985.7												
**TOP**	**300**	4	0.07	**±**	0.01	3985.7												
**ZON**	**300**	5	0.09	**±**	0.01	3033.3												
**PRC**	**300**	5	0.14	**±**	0.03	2007.7												
**GAB**	**300**	4	0.03	**±**	0.02	14700												
**MCL**	**100**	4	0.09	**±**	0.02	1011.1												
**NIA**	**100**	4	0.03	**±**	0.02	3233.3												
**DPR**	**300**	4	0.22	**±**	0.1	354.55												

Due to limitations in time resolution of our protocol, we assigned a value of 3s for carbamazepine (CBZ) and lidocaine (LID) whose onset time constants were too short to be resolved. On the other hand, lifarizine (LIF) and flunarizine (FLR) were completely irreversible within the duration of the protocol (200 s of washout); we assigned the value 500 s as the offset time constant of these drugs.

Abbr.:

a concentration,

b inhibition (inhibited fraction) at −90 mV holding potential using the 5 Hz train protocol,

c reversibility,

d use-dependence of the inhibition,

e drugs not potent enough for further investigation.

**Figure 1:**
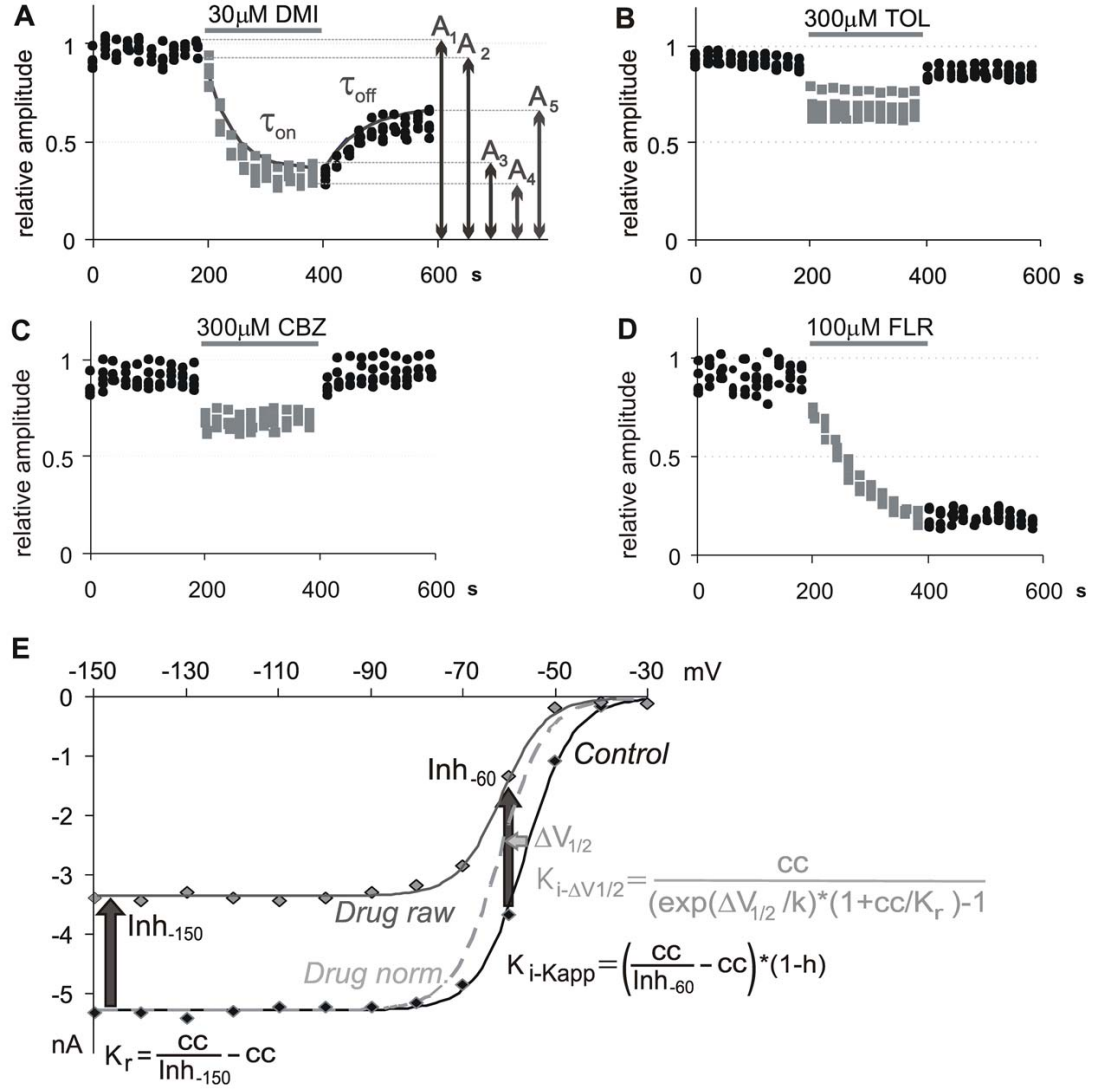
Calculation of parameters and examples for the different types of inhibition caused by SCIs. **A**)**–D**) Peak amplitudes of evoked currents (5 Hz trains of 5 depolarizations from −90 to −10 mV) are plotted against time. Black dots: Control. Grey dots: Drug perfusion. **A**) ‘**Type 1**’ inhibition (high potency, slow kinetics, partial reversibility, use-dependence). Calculation of properties of inhibition is illustrated. Inhibition: ***Inh*** *=  (A_1_−A_3_)/A_1_*; ***IC_50_*** =  (1−Inh) * cc/Inh, where “*cc*” is the concentration; Reversibility*:* ***Rev*** *=  A_5_/A_1_*; Use-dependence: ***UD*** *=  (A_3_/A_4_)/(A_1_/A_2_)*; τ**_on_** and τ**_off_** are determined by monoexponential fitting of peak amplitudes of the first evoked current in each train. **B)** Use-dependent ‘**Type 2**’ inhibition (low potency, fast kinetics, good reversibility, use-dependence). **C**) Non-use-dependent ‘**Type 2**’ inhibition (low potency, fast kinetics, good reversibility, no use-dependence). **D**) ‘**Type 3**’ inhibition (high potency, very slow kinetics, apparently irreversible, no use-dependence). **E**) Calculation of K_r_ and K_i_ values from steady-state availability curves.

We measured inhibition (Inh; from which we calculated IC_50_ values as described in Materials and Methods), reversibility of inhibition (Rev) and use-dependence (UD), as well as time constant of onset (τ_on_) and offset (τ_off_). The calculation of IC_50_, quantification of Rev and UD, and the determination of time constants are illustrated in Figure 1A. Because τ_on_ should be concentration-dependent if we assume a single binding reaction, we excluded it from the analyses. Reversibility measured by the QPatch instrument did not match reversibility values obtained by classic patch clamp (data not shown). It has been observed that washout of lipophilic compounds is delayed in automated electrophysiology platforms (e.g. [25]). However, reversibility even under these experimental conditions is a useful source of information regarding the physicochemical properties of drugs. Comments on the interpretation of these properties (Rev, τ_on_ and UD) are in Results S1.

Standard steady-state inactivation protocols were used to measure the shift of steady-state availability curve caused by the drugs. Currents were evoked by a 10 ms test pulse to −10 mV, preceded by 400 ms pre-pulses from −150 to −30 mV. The holding potential was −120 mV. Because the inhibition at hyperpolarized potentials (Inh_−150_), and the shift of the availability curves (ΔV_1/2_) are both concentration-dependent properties, we calculated affinities to hyperpolarized and depolarized states (“resting state affinity”: K_r_, and “inactivated state affinity”: K_i_), which, on the other hand, are concentration-independent characteristics of individual drugs. Calculation of K_r_ and K_i_ values are described in Materials and Methods, and illustrated in Figure 1E. State-dependence (SD) was quantified as the ratio K_r_/K_i_. Values of the eight properties for individual drugs are shown in Table 2 and Table 3. The calculated K_r_ and K_i_ values can be compared to data from the literature in Table 3.

**Table 3:** Values obtained from the steady-state inactivation protocol, and the calculated biophysical properties.

Drug	cc a	n	Inh b	±	SEM	ΔV_1/2_	±	SEM	K_r_	K_i−ΔV1/2_	K_i−Kapp_	SD c	K_r−Lit_	K_i−Lit_
code	µM					mV			µM	µM	µM		µM	µM
**A N T I D E P R E S S A N T S**														
**FLX**	**30**	4	0.13	±	0.02	−10.1	±	2.87	210.5	4.08	6.05	34.8	89.35	1.2
**SRT**	**30**	4	0.35	±	0.02	−15	±	1.01	56.41	1.47	2.1	26.9	NDA	∼1.7
**PRX**	**30**	5	0.55	±	0.09	−11.4	±	3.77	24.19	1.99	0.73	33	17	1.45
**AMI**	**10**	3	0.08	±	0.03	−11.3	±	2.47	133.9	1.45	3.34	40.1	38.11	0.52
	**30**	3	0.20	±	0.02	−11.2	±	0.76	124.9	3.69	4.92	25.4		
	**100**	3	0.59	±	0.05	−18	±	1.89	69.62	1.65	2	34.8		
**IMI**	**30**	4	0.25	±	0.08	−9.37	±	2.89	107.2	3.82	5.98	17.9	26.46	0.89
**DMI**	**30**	4	0.35	±	0.04	−5.87	±	0.53	57.03	8.06	4.37	13.1	29.24	1.15
**MPR**	**10**	4	0.12	±	0.03	−4.11	±	0.88	89.66	5.63	3.17	28.3	18.33	1.45
**NIS**	**100**	4	0.34	±	0.02	−6.68	±	0.52	194.2	19.1	7.1	27.3	NDA	NDA
**MIA**	**30**	4	0.18	±	0.03	−11.4	±	1.86	145.9	4.5	4.99	29.2	190	1.3
**MRZ**	**100**	4	0.24	±	0.01	−12.8	±	0.20	316.8	5.63	7.62	41.6	NDA	NDA
**BPR**	**100**	4	0.16	±	0.04	−6.97	±	1.27	603.6	26	25.5	23.6	NDA	NDA
**VFX**	**100**	5	0.11	±	0.02	−4.66	±	1.11	888.7	59.5	63.4	14	8	0.64
**NFZ**	**30**	4	0.09	±	0.03	−17.4	±	1.75	567.4	0.87	0.92	616	NDA	NDA
**TRZ**	**100**	4	0.19	±	0.04	−9.32	±	1.74	452	16.6	14.3	31.7	∼1000	∼111
**A N T I P S Y C H O T I C S**														
**HAL**	**30**	5	0.22	±	0.03	−4.72	±	1.24	113.5	13.8	2.96	38.4	7	0.28
**CPM**	**30**	7	0.32	±	0.05	−18.4	±	0.69	69.64	0.29	0.82	85.4	3.18	0.19
**CHX**	**30**	4	0.20	±	0.05	−15.8	±	2.18	128.5	0.73	1.74	74.1	NDA	NDA
**CLZ**	**100**	4	0.29	±	0.02	−8.35	±	1.42	248.6	11	12.4	20.1	NDA	NDA
**A N T I C O N V U L S A N T S**														
**CBZ**	**300**	4	0.21	±	0.02	−11.4	±	0.19	1169	39.9	26.4	44.3	946.1	21.8
**LTG**	**300**	4	0.12	±	0.01	−10.3	±	1.35	2267	31.3	36.7	61.8	1562	15.1
**DPH**	**300**	4	0.12	±	0.04	−4.93	±	1.25	2536	208.2	70.8	18.4	701.1	16.2
**L O C A L A N E S T H E T I C S/A N T I A R R H Y T H M I C S**														
**BPV**	**100**	6	0.14	±	0.01	−11.3	±	0.79	618.9	5.85	7.79	79.5	291	20.1
**LID**	**300**	4	0.09	±	0.01	−5.35	±	1.28	3192	89.6	65.7	48.6	686.7	17.7
**MEX**	**300**	4	0.32	±	0.03	−10.3	±	1.82	638.2	26.8	17.9	35.8	395.1	12.4
**FLC**	**300**	8	0.82	±	0.01	−12.4	±	0.94	64.69	7.9	5.7	11.3	354.9	5.55
**M I S C E L L A N E O U S**														
**RAN**	**300**	4	0.42	±	0.11	−5.52	±	1.00	435.5	66.8	10.7	40.7	1000	75
**MEM**	**100**	6	0.29	±	0.02	−1.77	±	0.54	242.2	109.8	28.5	8.5	178	6.67
**RIL**	**30**	3	0.08	±	0.01	−15	±	0.47	373.6	2.17	3.43	108.8	62.4	0.49
	**100**	3	0.22	±	0.01	−29.2	±	0.42	359.5	0.48	1.63	219.5		
**DIC**	**100**	4	0.03	±	0.01	−3.73	±	1.00	3119	81	87.84	35.5	784.9	6.5
**RIT**	**30**	5	0.13	±	0.02	−7.28	±	0.66	205.7	7.1	6.13	33.6	NDA	NDA
**AMB**	**100**	4	0.33	±	0.01	−9.87	±	0.90	200.9	12.2	17.6	11.4	110	8.17
**SIL**	**100**	4	0.43	±	0.03	−7.66	±	0.86	132.8	14	6.7	19.8	340	34.9
**TOL**	**100**	4	0.21	±	0.02	−6.47	±	1.74	391.1	30.9	21.1	18.6	384.3	5.07
**FLR**	**10**	5	0.04	±	0.00	−9.59	±	1.33	241	2	1.75	137.4	NDA	<0.2
**LIF**	**1**	4	0.02	±	0.00	−4.39	±	0.50	55.77	0.92	0.5	111.5	10.16	0.19

Measurements with different drug concentrations (e.g. amitryptiline and riluzole) resulted in similar K_r_ and K_i_ values, which confirmed the reliability of the calculations. The table shows geometric mean of individual K_r_, K_i−ΔV1/2_ and K_i−Kapp_ data (arithmetic mean ± SEM values are shown in Results S7). K_r_ and K_i_ data from the literature were calculated and averaged as described in [19]. Averaged data are shown in columns K_r−Lit_ and K_i−Lit_, except for trazodone [42] and flunarizine [43].

Abbr.:

a concentration,

b inhibition (inhibited fraction) at holding potential −150 mV,

c state-dependence.

### Classification of drugs based on properties of inhibition

At least three distinct types of inhibition were identified by observing drug behavior during the 5 Hz train and the steady-state inactivation protocols, and the examples are shown in Figure 1A–D.

‘Type 1’ (Figure 1A) drugs had high potency (K_i_ 0.73 to 6.1 µM; IC_50_ 14 to 43 µM), slow onset and offset kinetics (time constants between 10 and 53 s), partial reversibility (between 0.2 and 0.6) and use-dependence (1.09 to 1.66). Drugs belonging to this type were mostly antidepressants: fluoxetine, sertraline, paroxetine, amitriptyline, imipramine, desipramine and maprotiline, as well as the antipsychotic haloperidol, and the anxiolytic ritanserin.

The properties of ‘Type 2’ drugs (Figure 1B and C) were low potency (K_i_ 17 to 88 µM; IC_50_ >95 µM), fast kinetics (time constants <27 s) and almost full reversibility (>0.75) Drugs belonging to this type were the three effective anticonvulsants (carbamazepine, lamotrigine, phenytoin), the Class IB antiarrhythmic lidocaine and mexiletine, as well as diclofenac, venlafaxine, tolperisone, bupropion, ambroxol and memantine. The group can be further divided: lidocaine, mexiletine, ambroxol and tolperisone (Figure 1B) were use-dependent (UD 1.14 to 1.39). Anticonvulsants, memantine, venlafaxine, bupropion and diclofenac (Figure 1C), on the other hand, showed no significant use-dependence (0.95 to 1.05).

A distinct group, ‘Type 3’, was formed by the neuroprotectants flunarizine and lifarizine (Figure 1D). These drugs had high potency, very slow kinetics, apparent irreversibility (no recovery within the 200 s of washout within this experimental environment) and no use-dependence.

Of the remaining 13 drugs 7 were between ‘Type 1’ and ‘Type 2’ (nisoxetine, clozapine, silperisone, mianserine, mirtazapine, ranolazine and trazodone; named ‘Type 4’), 2 compounds between ‘Type 1’ and ‘Type 3’ (the antipsychotics chlorpromazine and chlorprothixene), while the remaining 4 drugs, bupivacaine, flecainide, nefazodone and riluzole seemed to have their own specific type of inhibition: Bupivacaine with high use-dependence and state-dependence; flecainide, with high use-depencence but low state-dependence and low K_r_, indicating that it is an open channel blocker; nefazodone with an exceptionally high state-dependence, and riluzole with high state-dependence and no use-dependence.

**Figure 2:**
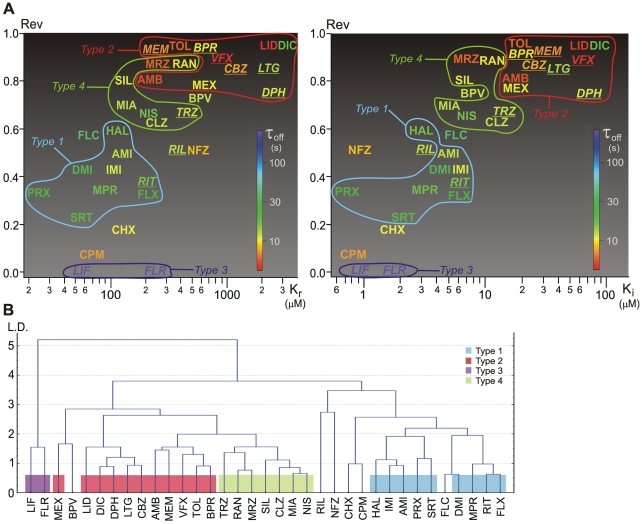
Localization of SCIs in the “biophysical space”. Clustering based on properties of inhibition. **A**) Distribution of the biophysical properties illustrated in a quasi-three-dimensional plot. Reversibility (Rev) values are plotted against K_r_ and K_i_ values, τ_off_ is color coded on a logarithmic scale. Lack of use-dependence is indicated by underlined italic fonts. Drugs classified into different types of inhibition are circled. The position of overlapping codes were minimally (<5%) adjusted for visibility. For exact values see Table 2 and Table 3. **B**) Result of a cluster analysis based on seven properties of inhibition: log K_r_, log K_i_, log IC_50_, log SD, UD, Rev and log τ_off_. (Weighted pair group average method was used as amalgamation rule, with Euclidean distance measure.) L.D.: Linkage distance.

Figure 2A illustrates quasi-three dimensional projections of drug locations in the eight-dimensional “biophysical space”. Rev values are plotted against K_r_ and K_i_ values (left and right panel, respectively), while the color indicates τ_off_. Non-use-dependent drugs are marked by underlined italic fonts. The types defined above are circled. Note how the relative position of drugs with a high SD changes: Flunarizine (FLR), chlorpromazine (CPM), chlorprothixene (CHX), riluzole (RIL), bupivacaine (BPV) and, most of all, nefazodone (NFZ) all move to the left on the Rev vs. K_i_ plot as compared to the Rev vs. K_r_ plot.

We also observed that for ‘Type 1’ and ‘Type 3’ drugs IC_50_ values were typically midway between K_r_ and K_i_, while for many ‘Type 2’ and ‘Type 4’ drugs IC_50_ was closer to K_r_ (for further discussion see Results S2).

In order to quantify differences and test the validity of the subjective classification, we performed a cluster analysis using the properties of inhibition. We took the logarithm of K_r_, K_i_, IC_50_, SD and τ_off_ values, and normalized all seven properties by subtracting the mean (of all drugs) from the values for individual drugs and dividing by the standard deviation. Results of the cluster analysis are shown in Figure 2B.

The overall picture represents the subjective description quite well. ‘Type1’ and ‘Type3’ inhibitions were clearly recognized, as well as the separateness of riluzole and nefazodone. Using different amalgamation rules and distance measures resulted in similar, although not identical classifications. The differences between our subjective classification and the result of cluster analysis were the following:

Flecainide, although had somewhat higher IC_50_, τ_off_ and Rev values, was consistently clustered into ‘Type 1’ group. However, despite the similar potency and kinetics, this compound has been shown to have a separate mode of action, being an open channel blocker [26].

‘Type 2’ and ‘Type 4’ groups were not clearly separated.

Bupivacaine was clustered into a separate subgroup of the ‘Type2’ – ‘Type4’ group together with mexiletine. However, its UD and SD values (1.62 and 79.4, respectively) were higher than the rest of either ‘Type 2’ or ‘Type 4’ compounds.

**Figure 3:**
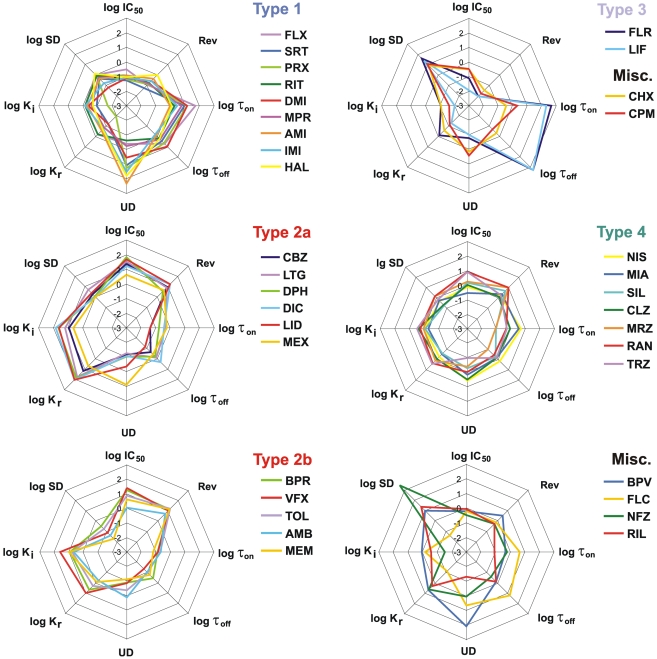
Properties of inhibition illustrated on radar diagrams. Different panels show different types of inhibition. ‘Type 2’ drugs were divided into two panels (based on the results of cluster analysis) for the sake of visibility. For individual values of properties see Table 2 and Table 3.

The biophysical properties of major groups are also shown in Figure 3. The shape of each radar diagram gives an impression of the actual type of inhibition. This way of illustration helps to judge the correctness of our initial classification, and the classification by cluster analysis.

Figure 2A also indicates that there was a strong correlation between certain properties of inhibition. We calculated correlation coefficients (for K_r_, K_i−Kapp_, K_i−ΔV1/2_, IC_50_, SD values and time constants we used logarithmic transformation). All properties significantly correlated with each other, with the exception of UD and SD. Use-dependence only correlated with potency (K_r_, K_i− Kapp_, K_i−ΔV1/2_ and IC_50_) values, while state-dependence only correlated with Rev and the two K_i_ values (Table 4), and notably showed no correlation with K_r_.

**Table 4:** Cross-correlation matrix for properties of inhibition (upper part) and correlations between properties of inhibition and chemical descriptors (lower part).

	log IC_50_	Rev a	log τ_on_	log τ_off_	UD b	log K_r_	log K_i−ΔV1/2_	log K_i−Kapp_	log SD c
log IC_50_	-	**0.82**	**−0.76**	**−0.59**	**−0.51**	**0.86**	**0.80**	**0.88**	−0.16
Rev a	**0.82**	-	**−0.79**	**−0.67**	−0.23	**0.67**	**0.83**	**0.82**	−*0.38*
log τ_on_	**−0.76**	**−0.79**	-	**0.85**	0.23	**−0.61**	**−0.55**	**−0.64**	0.16
log τ_off_	**−0.59**	**−0.67**	**0.85**	-	−0.02	−*0.41*	−*0.39*	**−0.51**	0.24
UD b	**−0.51**	−0.23	0.23	−0.02	-	**−0.49**	−*0.34*	−*0.38*	−0.11
log K_r_	**0.86**	**0.67**	**−0.61**	−*0.41*	**−0.49**	-	**0.68**	**0.80**	0.18
log K_i−ΔV1/2_	**0.80**	**0.83**	**−0.55**	−*0.39*	−*0.34*	**0.68**	*-*	**0.92**	**−0.48**
log K_i−Kapp_	**0.88**	**0.82**	**−0.64**	**−0.51**	−*0.38*	**0.80**	**0.92**	*-*	**−0.44**
log SD c	−0.16	−*0.38*	0.16	0.24	−0.11	0.18	**−0.48**	**−0.44**	-
MW d	**−0.44**	**−0.46**	**0.47**	**0.44**	0.07	−0.30	−*0.40*	**−0.49**	*0.36*
pKa e	**−0.54**	−0.30	0.30	0.04	**0.47**	**−0.68**	**−0.42**	**−0.46**	−0.25
logP f	**−0.74**	**−0.76**	**0.60**	**0.58**	0.14	**−0.53**	**−0.73**	**−0.67**	0.32
PSA g	*0.39*	0.24	−0.25	−0.11	−*0.35*	*0.39*	0.26	0.24	0.19
logD_7.3_ h	*−0.38*	***−*** **0.54**	*0.36*	**0.48**	−0.19	−0.06	***−*** **0.54**	***−*** **0.49**	**0.69**
Min.proj.area i	***−*** **0.55**	***−*** **0.49**	**0.55**	**0.43**	*0.33*	−*0.37*	−0.41	***−*** **0.50**	0.27
Aromatic AC J	***−*** **0.49**	***−*** **0.63**	**0.61**	**0.58**	−0.08	−0.21	**−0.48**	**−0.52**	**0.54**
log N(pKa) k	*0.41*	0.23	−0.22	−0.00	**−0.45**	**0.64**	0.24	0.18	**0.55**
	*p<0.05*				**p<0.01**				

Abbr.:

a reversibility,

b use-dependence,

c state-dependence,

d molecular weight,

e acidic dissociation constant,

f octanol-water partition coefficient,

g polar surface area,

h distribution coefficient at pH = 7.3,

i minimal projection area,

j aromatic atom count,

k N(pKa): the fraction of neutral molecules at pH = 7.3.

It is apparent that more potent drugs (whether potency was measured by K_r_, K_i− Kapp_, K_i−ΔV1/2_ or IC_50_) tended to have slower onset and offset kinetics, tended to be less reversible, and tended to be more use-dependent. High inactivated state affinity (low K_i_) predicted high state-dependence, while high resting affinity (low K_r_) did not.

In summary, we have identified at least three distinct types of inhibition, these may correspond with: i) different binding sites, ii) different access pathways, iii) different modes of action (channel block, stabilization of a non-conducting conformation, membrane-mediated inhibition, deformation of the channel by an induced fit mechanism, etc. - see [9]), iv) different binding kinetics (including kinetics of: partitioning into and out of the membrane phase, deprotonation and protonation, translocation between the two leaflets of the membrane, horizontal diffusion within the membrane and the actual entry to- and exit from the binding site).

The next logical question was, whether distinct types of inhibition, correspond with specific chemical properties, i.e., whether the type of inhibition can be predicted based on chemical structure.

### Relationship between chemical properties and biophysical properties of inhibition

We used the cheminformatics software JChem for Excel (see Materials and Methods) to generate possible numerical chemical descriptors (i.e., to calculate chemical properties from the chemical structure). The correlation matrix of the 58 descriptors is given in Results S3. The correlations helped to recognize informative descriptors and to detect redundancies.

It was even more important to analyze correlations between individual chemical descriptors and the eight properties of inhibition, because this showed which specific chemical properties affect individual aspects of inhibition. The complete analysis for all 58 descriptors is described in Results S4. Based on the correlations among chemical descriptors (Results S3), and the correlations between chemical descriptors and properties of inhibition (Results S4 and S5), we selected the descriptors which were most informative regarding the mode of action of SCIs (Results S4). Only these descriptors will be discussed in the following section (values for individual drugs are shown in Results S6).

**Figure 4:**
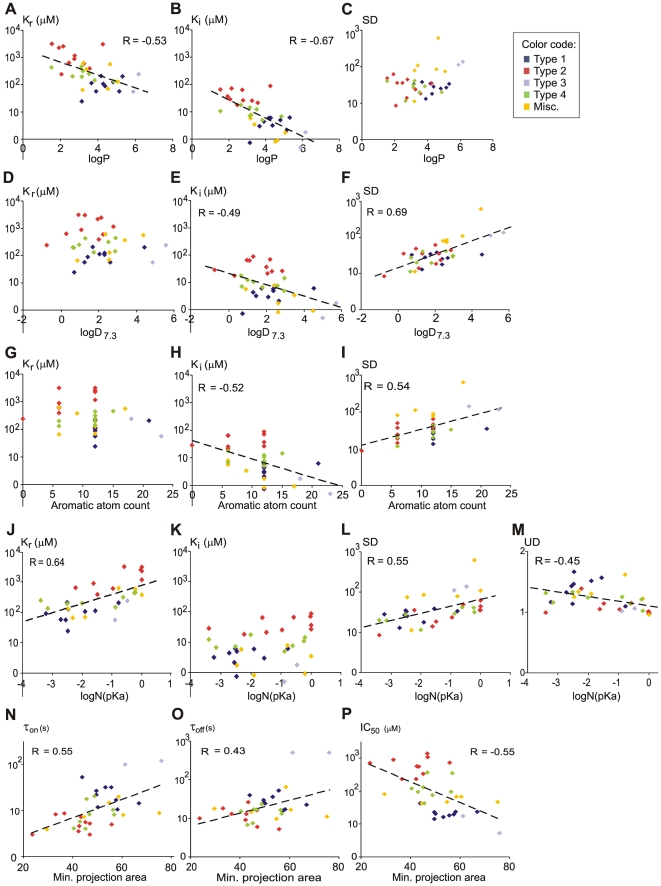
Dependence of properties of inhibition on specific chemical properties. Drugs belonging to different types of inhibition are shown in different colors. For values of individual drugs see Table 2, Table 3 and Results S6. Biophysical properties plotted against chemical descriptors. **A**)**–C**): logP, **D**)**–F**): logD_7.3_, **G**)**–I**): aromatic atom count, **J**)**–M**): logN(pKa), **N**)**–P**): minimal projection area. Regression lines and correlation coefficients (R) are only shown where the correlation was significant (p<0.01).

The single most important chemical property that determined potency (K_i_, and IC_50_) values, as well as reversibility of drugs, was logP, as seen in Figure 4A–B (R = −0.53, −0.67, −0.74, and −0.76 for K_r_, K_i_, logIC_50_ and Rev, respectively) and in Results S4. The correlation values of logD_7.3_ (the distribution coefficient at pH = 7.3) were considerably lower but still significant, except for K_r_ (R = −0.06, −0.49, −0.38, and −0.54 for K_r_, K_i_, logIC_50_ and Rev, respectively), which indicates that K_r_ was not influenced by logD_7.3_ (Figure 4D–E).

This suggests that the binding site and/or the access pathway is separated from the extracellular environment, and in this local milieu drug molecules are deprotonated. Lipophilicity of the positively charged form seemed to be indifferent for the resting binding site, but for the inactivated binding site it was important: drugs which are strongly lipophilic even in their protonated form were more potent against inactivated conformation.

Indeed, the property that showed the highest correlation with logD_7.3_ was state-dependence (Results S4). This means that the best predictor of high state-dependence for a drug was lipophilicity of the dominant form of the molecule at pH = 7.3, even though this is the positively charged form for 27 out of the 35 molecules (Figure 4F). Lipophilicity of the neutral form did not predict high state-dependence (Figure 4C).

The next best predictor of state-dependence was aromaticity (aromatic atom count, aromatic ring count, aromatic bond count). These descriptors correlated significantly with SD, K_i_ and IC_50_ (R = 0.54, −0.52, −0.49, respectively for aromatic atom count), but not with K_r_ (R = −0.21) (Figure 4G–I; Results S4). Measures of aromaticity were also good predictors of reversibility and time constants.

The most important determinant of K_r_ was pKa, the correlation (R = −0.68) was higher than with logP (R = −0.53). Molecules with high pKa values, i.e., which are predominantly positively charged at physiological pH, were more potent against resting channels, while inactivated affinity did not correlate with either pKa or N(pKa). (Because the distribution of pKa values was rather skewed, for the purpose of illustration, neutral fraction at pH = 7.3 (designated as ‘N(pKa)’) was calculated from pKa values as described in Materials and Methods. Because most drugs were either neutral or positively charged at pH = 7.3, N(pKa) is a good substitute for pKa, with the only exceptions of phenytoin and diclofenac, which have negatively charged forms (6.5% and 99.95%, respectively) at pH = 7.3.) The correlations are shown in Figure 4J–K. Interestingly, predominantly neutral molecules were more state-dependent than positively charged ones (Figure 4L). This is in contrast to the general view, that potent SCIs should be predominantly positively charged molecules (see Introduction). On the other hand, use-dependence was more prominent among positively charged drugs (Figure 4M). Another descriptor that showed significant (but only at p<0.05 level) correlation with both K_r_ and UD (but not with K_i_), was polar surface area (PSA) (R = 0.39 and −0.35, respectively). Small polar surface area corresponded with higher resting affinity and higher use-dependence.

Time constants were determined by aromaticity, lipophilicity and the size of the molecule: large, lipophilic molecules with multiple aromatic rings had slower onset/offset kinetics. From the descriptors which quantify different aspects of molecular size, minimum projection area was found to show somewhat higher correlation than molecular weight (Figure 4N–O). Larger molecules were also found to be more potent (Figure 4P), which may partly be due to the fact that larger molecules are also more lipophilic.

In summary, we have identified chemical descriptors which predict K_r_, K_i_, IC_50_, state-dependence, reversibility, use-dependence, onset and offset time constants. While logP is essential for both resting and inactivated state affinity, resting affinity is higher for positively charged drugs, and interestingly, the lipophilicity of this charged form does not seem to be important. One explanation may be that this type of inhibition requires both interaction of the neutral form with the membrane, and interaction of the charged form with an aqueous environment. On the other hand, for inactivated state affinity positive charge is not essential, in fact neutral molecules tend to show higher state-dependence, furthermore lipophilicity of both the neutral and the charged forms are important, as well as aromaticity. This may suggest that the immediate environment of the bound molecule is apolar, and binding probably involves π - π interactions with aromatic residues (see discussion).

### Classification of drugs based on chemical properties

**Figure 5:**
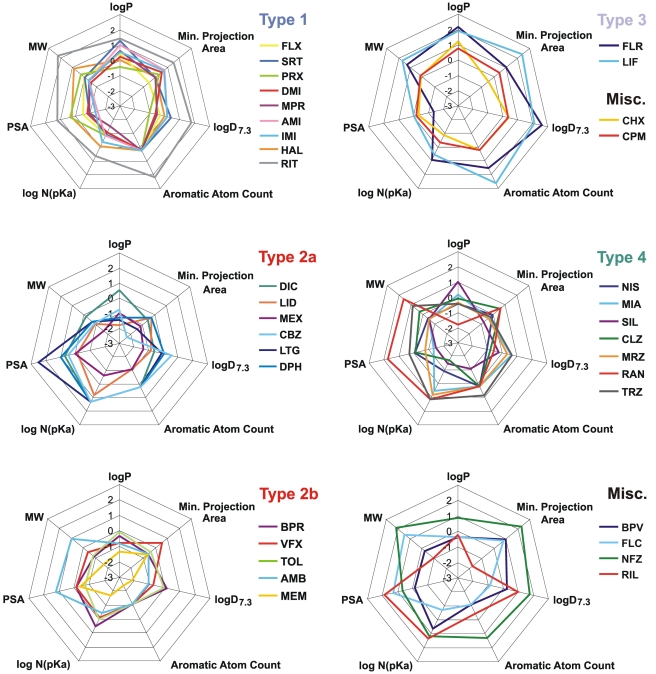
Selected chemical descriptors of drugs illustrated on radar diagrams. Different panels show different types of inhibition. ‘Type 2’ drugs were divided into two panels as in Figure 3, for the sake of visibility. For individual values of properties see Results S6.

We have investigated whether the type of inhibition can be predicted from chemical properties of the molecule. We have illustrated major chemical properties of the drugs in radar diagrams (Figure 5). It is apparent from the figure, that ‘Type 1’, ‘Type 3’ groups, as well as the anticonvulsants of ‘Type 2’ form definite, compact regions within the “chemical space”, while the regions which contain the rest of ‘Type 2’, and ‘Type 4 drugs are rather indefinite, and contain compounds which are chemically diverse.

**Figure 6:**
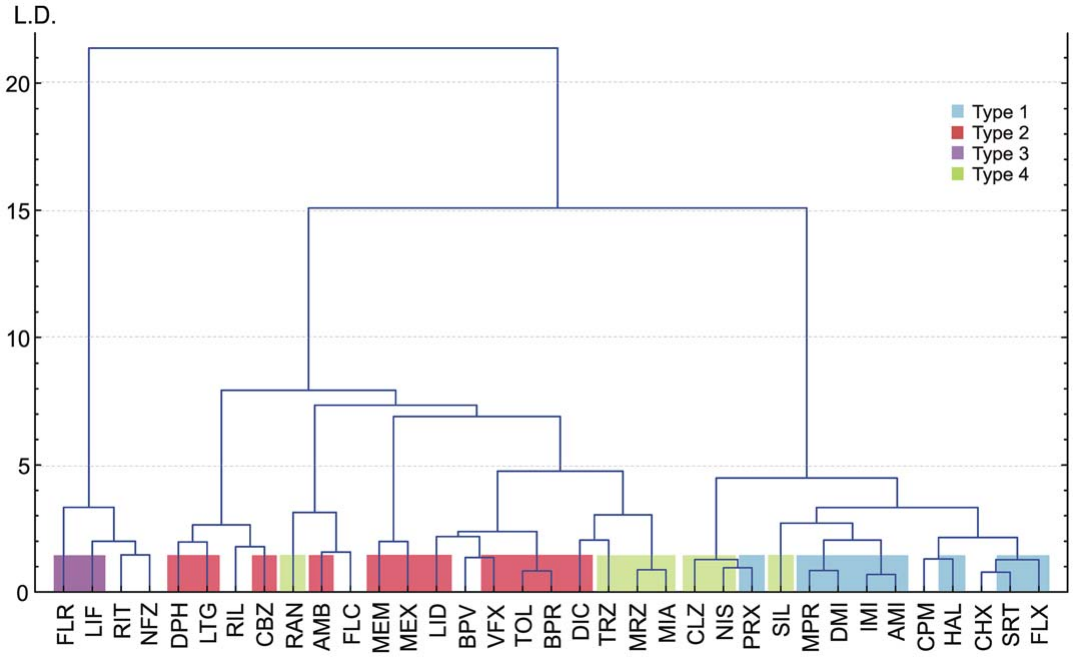
Clustering based on selected chemical descriptors. Result of a cluster analysis based on seven chemical descriptors: logP, logD_7.3_, MW, polar surface area, aromatic atom count, minimal projection area and log N(pKa). (Ward’s method was used as amalgamation rule, with Euclidean distance measure.) L.D.: Linkage distance.

A cluster analysis of drugs based on 7 chemical descriptors (molecular weight, minimum projection area, logP, logD_7.3_, log N(pKa), polar surface area, and aromatic atom count) was performed (Figure 6).

One of the three major groups contained ‘Type 3’ drugs together with ritanserin and nefazodone, the second group all ‘Type 2’ drugs, and the third one contained all ‘Type 1’ compounds except ritanserin. ‘Type 4’ drugs, which produced inhibition with properties between ‘Type 1’ and ‘Type 2’ were classified together with either of these groups, and unclassified drugs, as expected, were heterogenous, and were scattered among the three major clusters.

The similarity between clustering based on biophysical or chemical properties is obvious, which allows prediction of biophysical properties with a considerable certainty. Within certain sections of the “chemical space” (such as those populated by ‘Type 1’, ‘Type 3’ compounds, or anticonvulsants from the ‘Type 2’ group; see Figure 5), specific inhibition types are obviously predominant (may even be exclusive). Predominantly positively charged (>98.75%; pKa 9.1 to 10.5) compounds with two aromatic rings, high logP (3 to 5.2), MW between 260 and 330, and PSA less than 45 Å^2^ constituted the group of ‘Type 1’ drugs (without ritanserin). Anticonvulsant SCIs (a subset of ‘Type 2’ drugs), on the other hand, are predominantly neutral (>93.5%; pKa<4.5), have relatively low logP (1.9 to 2.8), MW between 230 and 260, and PSA between 46 and 91 Å^2^. Finally ‘Type 3’ drugs have three or four aromatic rings, a very high logP (5.8 to 6.2), high MW (400 to 440), moderate pKa (7.5 to 8.2) and low PSA (<37 Å^2^). It is yet to be verified experimentally whether other compounds beyond the ones investigated in this study, which fall within these sections of the “chemical space” are necessarily SCIs, and whether they produce the expected type of inhibition.

## Discussion

### Classification of SCIs by automated patch clamp using multiple properties of inhibition

SCIs are currently developed intensively for several indications, such as pain syndromes, epilepsy, ischemia and neurodegenerative diseases.

For drug development it is important to know the binding site as thoroughly as possible. It is often assumed that sodium channels possess a single drug binding site (the “local anesthetic receptor”), however, several experimental data indicate that alternative binding sites and alternative modes of action do exist. From the 35 drugs investigated in this study 10 has been studied on mutant channels. Lidocaine, mexiletine and ranolazine seem to share the binding site; for amitriptyline, lamotrigine, phenytoin, flecainide and bupivacaine the binding site seem to partially overlap with the local anesthetic binding site; and for sertraline and paroxetine an entirely different binding site seems to exist [9]. Although a comparative study of a number of SCIs on mutant channels would be a much needed endeavor, in this study we did not directly study possible binding sites by mutagenesis. Instead we attempted to perform a classification of SCIs using wild type channels. This approach is unable to prove alternative binding sites, but it is able to pinpoint drugs or groups of drugs with specific modes of action, which are worth studying with other approaches. We believe that the results of this classification will give directions to later studies of different binding sites.

We found that SCIs are diverse in their potency, kinetics, reversibility, use-dependence and state-dependence. We described three major types of inhibition, and besides we identified a number of compounds (e.g., nefazodone, riluzole, flecainide) which have additional distinct modes of action.

### Prediction of properties of inhibition using chemical descriptors

We identified specific chemical descriptors which predict particular properties of inhibition.

Partition coefficient (logP) was a major determinant of potency, kinetics and reversibility. Lipophilic molecules tended to be more potent in terms of K_r_, K_i_ and IC_50_ (measured at -90 mV, using the 5 Hz train protocol) values; in addition the inhibition was less reversible, with slower onset and offset kinetics. This is in accordance with studies on structure-activity relationships, where a linear relationship between logP and logIC_50_ values were described based on the inhibition of action potentials [17], [27] and of [^3^H]BTX binding [15], [16], [18]. A similar relationship between lipophilicity and potency has been shown for a number of other transmembrane proteins [28], [29], [30].

Explanation for the correlation between logP and potency can be either of the following two possibilities: (1) SCIs must cross the membrane in order to enter a hydrophilic environment (which may be the intracellular fluid or the binding site itself); therefore, in order to be able to cross the membrane, the drugs must be lipophilic. (2) The binding site itself is hydrophobic. The first explanation supposes that drug molecules have to partition into the membrane, and then out of the membrane toward the intracellular fluid or the binding site. Too high lipophilicity would prevent effective partitioning out of the membrane environment; therefore logP should have a definite optimum. Although it cannot be statistically proven from our data, note that in Figure 2A the mean of K_r_ values (left panel) for ‘Type 1’ drugs is smaller than the mean K_r_ of the more lipophilic ‘Type 3’ drugs, while for K_i_ values (right panel), more lipophilic ‘Type 3’ drugs are more potent than the average of ‘Type 1’ drugs. This may suggest that lipophilic interactions (logP and logD) are more important in inactivated state affinity.

Among the eight properties of inhibition state-dependence (i.e., K_r_/K_i_ ratio) is of particular importance. It is thought to be essential for the safety of SCIs: state-dependent drugs are able to selectively inhibit excessive/pathological firing patterns, such as in epilepsy, neuropathic pain or cardiac arrhythmia, while their effect on normal firing activity is minimal.

Distribution coefficient at pH = 7.3 (logD_7.3_) was the best predictor of state-dependence, and one of the best predictors of K_i_. Drugs with high logD_7.3_ were more state-dependent, and had high inactivated affinity, while this property was irrelevant for resting affinity (see Figure 4D and E). There are two major reasons why logD_7.3_ of a drug can be high: Some of the compounds had a logP so high (>4.0), that even in their charged form they were still strongly lipophilic (some examples are fluoxetine, sertraline, amitriptyline, imipramine, haloperidol, chlorpromazine, chlorprothixene and silperisone). Some other compounds had a low pKa value (<8.2), which means that a considerable fraction (>10%) of the molecules is neutral at pH = 7.3, therefore there is not much difference between logP and logD_7.3_ (less than 1 unit). Some examples are carbamazepine, lamotrigine, phenytoin, mirtazapine, trazodone, bupivacaine and ranolazine. Finally, there were five drugs, where both the logP was high and the pKa was low: nefazodone, riluzole, ritanserin, flunarizine and lifarizine. These drugs (with the exception of ritanserin) had the highest state dependence values among the 35 drugs investigated.

From this reasoning it follows, that the ratio of neutral form, N(pKa) also had to be a major determinant of state-dependence. Indeed, less charged molecules were found to be more state-dependent. This is contrary to the widely held view that SCIs should be positively charged. Because there was no significant correlation between N(pKa) and logD_7.3_ (neutral molecules did not have higher logD_7.3_ values), the ratio of neutral molecules probably affects state-dependence directly.

On the other hand, if we consider resting affinity, we can notice that positively charged SCIs tended to be more potent. The finding, that positively charged molecules are better inhibitors of resting channels irrespectively of the lipophilicity of the charged form (K_r_ did not depend on logD_7.3_), suggests that in resting inhibition an interaction of the charged form and the channel occurs within a polar environment. The major determinant of resting affinity was found to be pKa, indicating the importance of positive charge in resting inhibition. It was also the most important determinant of use-dependence, which is in accordance with previous studies, where positively charged molecules had slower kinetics and showed more use-dependence [4], [17], [20], [21], [31], [32].

The third major determinant of state-dependence is aromaticity. While resting affinity was not dependent on the number of aromatic rings, inactivated affinity showed a definite dependence. This suggests that interactions between aromatic rings (π-π interactions) are important in binding to inactivated state. Aromaticity also determined time constants and reversibility. This may be one explanation of the finding that dimers of lidocaine (containing two aromatic rings) show both increased potency and decreased reversibility [33].

The role of aromaticity of the most important residue of the local anesthetic binding site (Phe1710 in rNav1.3) in use-dependent- but not resting inhibition has been shown by different amino acid substitutions. For resting inhibition hydrophobicity of the residue was sufficient, while effective use-dependent inhibition required that the residue was aromatic [34]. The role of the aromatic ring has also been investigated by using unnatural derivatives of phenylalanine (Phe1579 in rNav1.4) in which the π electron clouds were distorted. This affected use-dependent inhibition, and recovery from inactivated state, but left tonic inhibition (resting affinity) intact [10]. These results could be explained by either cation-π or π-π interactions. Our results support the role of π-π interaction, which is consistent with single channel analysis of the inhibition by local anesthetics; two distinct types of inhibition were observed at single channel level: rapid block (manifested as decreased single-channel conductance), and discrete block (appearance of distinct closed periods). The former could be reproduced by the charged amine fraction of local anesthetics, while phenol, which resembles the aromatic part of local anesthetics, caused discrete block [35]. Mutation of the phenylalanine residue (Phe1579 in rNav1.4) abolished discrete block, while not affecting rapid block [12].

In summary, we propose that drugs with high state-dependence are more likely to be found among compounds which contain more than one aromatic rings, and which have logD_7.3_ >3.0, and pKa <8.0 (conformity with all three conditions is not absolutely necessary). We have identified a couple of highly state-dependent compounds, which could be used as a basis for further drug development. The SCI property of bupivacaine, riluzole, flunarizine and lifarizine are well known, but we would like to call attention to the attractive properties of nefazodone, chlorpromazine and chlorprothixene, as highly state-dependent SCIs.

Our data confirm our previous results based on a meta-analysis of the literature [19]. We attempted to find chemical properties which predict resting and inactivated affinity. The advantage of that study was the larger pool of data (139 compounds), which theoretically should help identification of correlations. However, the diversity of preparations, experimental protocols and analysis methods seriously compromised comparability. We could detect the role of logD_7.3_, and aromaticity in determining K_i_, but the correlations were less convincing. Furthermore, the role of positive charge in resting affinity, and neutrality in inactivated affinity could not be detected. The advantages of using identical experimental conditions for all drugs – as in our current study – are: improved reliability of data, the possibility of correlating chemical properties with multiple biophysical properties, and the possibility of detecting distinct types of inhibition within the “multi-dimensional space” defined by both biophysical properties of inhibition and chemical properties of the molecules.

### What is the significance of identification of different types of inhibition?

Therapeutic applicability is not determined solely by the potency. In fact some of the most widely used drugs (lidocaine, phenytoin, carbamazepine) are among the least potent SCIs. However, we expect that SCIs acting by similar mechanisms will have similar therapeutic action (provided that sodium channel inhibition is the principal element in its effect). In this respect it is important to find out about different types of inhibition, to locate novel drugs in the “biophysical space”, and to learn how this location is determined by chemical properties. This study, of course, have not accomplished mapping of the entire “biophysical space” for SCIs, but we hope the concept has been introduced, and at least we have identified three basic types of inhibition and a couple of additional drugs with interesting properties. Antiarrhythmics and local anesthetics (bupivacaine, lidocaine, mexiletine and flecainide) were a diverse group, while the three anticonvulsants (carbamazepine, phenytoin, lamotrigine) were found to be similar. A group of antidepressants: selective serotonin reuptake inhibitors, tricyclic antidepressants and maprotiline formed a fairly homogenous group, while the remaining antidepressants were diverse in both chemical and biophysical properties. It is worth noting that flunarizine and lifarizine (neuroprotective agents [36]) occupy a specific area in both “biophysical space” and “chemical space”.

### Summary and conclusions

In summary, we have recorded multiple parameters of inhibition, which did not make our measurement more costly or time consuming but provided us with additional information. With this extra information, we established that SCIs are heterogeneous, delineated specific types of inhibition, and with the help of chemical descriptors identified specific predictors of state-dependence. The protocols used in this study were fairly simple; the accuracy of the method can be further improved by including measurements for additional biophysical parameters (e.g. frequency-dependence, pH-dependence, etc). The challenge is to maximize the information content that can be obtained from more complex protocols, while not increasing the cost of measurements, and keeping the analysis manageable.

We believe that this new approach of mapping drugs in the “biophysical space”, rather than determining a single IC_50_ value will help drug discovery, especially if we can determine the specific chemical properties which predict individual types of inhibition. This concept may be particularly profitable in the study of certain ion channels, which are notorious of their promiscuity in drug binding.

## Materials and Methods

### Cell cultures

HEK-293 cells stably expressing rNav1.2 sodium channels were obtained from NeuroSearch (Ballerup, Denmark). The cells were grown in Dulbecco's modified Eagle medium (catalog no. 32430-027, Invitrogen) supplemented with 10% FBS. Prior to use, the cells were trypsinized (catalog no. 15400-054, Invitrogen) and subsequently kept in suspension in the QPatch cell storage facility in CHO-S- SFM-II medium (catalog no. 12052-114, Invitrogen).

### Solutions and drugs

Cells were automatically prepared for application to the chips (centrifuged and washed twice, then resuspended in extracellular solution) as described previously [37]. Composition of the extracellular solution was: (in mM): 140 NaCl, 3 KCl, 1 CaCl_2_, 1 MgCl_2_, 0.1 CdCl_2_, 20 TEA-Cl, 5 HEPES, adjusted to pH 7.3, Osmolality: ∼320 mOsm. The intracellular solution consisted of the following (in mM): 135 CsF, 10 NaCl, 1 EGTA, 10 HEPES, adjusted to pH 7.3 with CsOH (∼5 mM), Osmolality: ∼320 mOsm.

Drugs were obtained from Sigma, Tocris, or synthesized in Gedeon Richter Plc. (Budapest, Hungary). The list of drugs together with the three-letter codes, as well as their therapeutic indication, main mechanism of action, and human plasma concentration are shown in Table 1. Source of drugs and preparation of stock solutions are shown in Materials and Methods S1.

### Electrophysiology

All electrophysiological experiments were conducted on Qpatch-16 or QPatch HT instruments using QPlate™ chips. For a detailed description of the instrument and the patch-clamp chips see [37], [38]. Data were sampled at a frequency of 25 kHz and filtered at 5 kHz. Junction potential was calculated to be −11 mV and was corrected for. The amplifier was controlled and the data were collected by the Sophion QPatch client software. For initial data analysis the QPatch software was used. Built-in amplifiers provide an improved method for series resistance compensation. Leak subtraction (based on a standard P/n protocol) was used to subtract the capacitive component of currents.

Chip properties, distribution of gigaseal properties and stability of whole-cell parameters were essentially the same as described before for HEK-293 cells [37], [39]. Distribution of whole-cell membrane resistance, series resistance, whole-cell capacitance values and current amplitudes, as well as voltage-conductance curves for activation and inactivation with V_1/2_ values and slope factors are shown in Materials and Methods S2.

#### Analysis of data from the 5 Hz train protocol

From this protocol we extracted five parameters, as illustrated in Figure 1A. We calculated inhibition of a single “cc” concentration: *Inh  =  (amplitude_ctr_ – amplitude_drug_)/amplitude_ctr_*. IC_50_ values were estimated using the rearranged Hill equation assuming one-to-one binding: IC_50_  =  (1−Inh) * cc/Inh. Reliability of the calculation was verified by measuring the effect of different concentrations of the same drug (in the case of 5 out of the 35 drugs). The process is illustrated using the example of lidocaine in Materials and Methods S3.

#### Analysis of data from the steady-state inactivation protocol

Resting affinity (K_r_) values were calculated from the inhibition at −150 mV pre-pulse potential (Inh_−150_), using the rearranged Hill equation as described above (Figure 1E). Inactivated affinity (K_i_) values were calculated assuming a simple four-state model of drug binding and channel gating [40], using two separate methods: (1) from the shift of the availability curve (ΔV_1/2_), the slope of the Boltzmann fit of the availability curve (k) and K_r_, using the formula described by Bean, Cohen and Tsien [40] (K_i−ΔV1/2_; see Figure 1E), or (2) from the apparent affinity (K_app_) at a certain pre-pulse potential, which was calculated from the inhibition (e.g. Inh_−60_) as described above, and the availability (h) at the same pre-pulse potential, using the formula from Kuo and Bean [41] (Figure 1E). Differences between the two calculations of K_i_ were minimal, K_i−ΔV1/2_/K_i−Kapp_ ratios were 0.98±0.098. For further analysis K_i_ values obtained with the latter method were used, because these seemed more reliable (and they could be verified at different holding potentials). Geometric means of measured K_r_, K_i−ΔV1/2_ and K_i−Kapp_ values are shown in Table 3. (Arithmetic means and SEM values are given in Results S7.)

### Cheminformatics

Chemical descriptors were generated using JChem for Excel 1.1.1 and Marvin 5.2 software from ChemAxon (Budapest, Hungary). Neutral fraction of drugs at pH = 7.3 was calculated using the rearranged Henderson-Hasselbalch equation: N(pKa) = 10^pH^/(10^pH^+10^pKa^).

### Data analysis and statistics

Exponential fitting was made using Origin 8 (Originlab, Northampton, MA). Statistical analysis was performed using Statistica 8.0 (StatSoft, Inc., Tulsa OK).

## Supporting Information

Materials and Methods S1 **Source of drugs and stock solutions.**
(PDF)

Materials and Methods S2 **Main electrical properties of cells.**
(PDF)

Materials and Methods S3 **Reliability of IC_50_ calculation from single-concentration inhibition values.**
(PDF)

Results S1 **Comments on biophysical properties of inhibition.**
(PDF)

Results S2 **Detailed discussion of state-dependence.**
(PDF)

Results S3 **Cross-correlations of 58 chemical descriptors, based on the chemical properties of the 44 drugs we used.**
(PDF)

Results S4 **Classification of 58 chemical descriptors based on their correlations with individual properties of inhibition.**
(PDF)

Results S5 **Correlation coefficients between chemical descriptors and biophysical properties.**
(PDF)

Results S6 **Values of selected chemical descriptors for the drugs studied.**
(PDF)

Results S7 **Geometric and arithmetic mean values of K_r_, K_i−ΔV1/2_ and K_i−Kapp_.**
(PDF)
