# Supplementary material for: Classification of Drugs Based on Properties of Sodium Channel Inhibition: A Comparative Automated Patch-Clamp Study
Source: PLoS One. 2010 Dec 20;5(12):e15568. doi: 10.1371/journal.pone.0015568 (PMC3004914; doi:10.1371/journal.pone.0015568)
Supplement: Materials and Methods S1 — Source of drugs and stock solutions. (PDF) [file pone.0015568.s001.pdf]

Materials and Methods S1 - Source of drugs and stock solutions.

	Drugs	Code	Company	Solvent	Stock (mM)
ANTIDEPRESSANTS	Fluoxetine hydrochloride	FLX	Sigma	water	10
	Sertraline hydrochloride	SRT	Sigma	DMSO	50
	Paroxetine hydrochloride	PRX	Richter	DMSO	10
	Amitriptyline hydrochloride	AMI	Sigma	water	10
	Imipramine hydrochloride	IMI	Sigma	water	10
	Desipramine hydrochloride	DMI	Sigma	water	10
	Maprotiline hydrochloride	MPR	Sigma	water	10
	Nisoxetine hydrochloride	NIS	Sigma	ethanol	10
	Mianserin hydrochloride	MIA	Sigma	ethanol	10
	Mirtazapine	MRZ	Tocris	DMSO	10
	Bupropion hydrochloride	BPR	Sigma	water	10
	Venlafaxine hydrochloride	VFX	Tocris	water	10
	Nefazodone hydrochloride	NFZ	Tocris	DMSO	10
	Trazodone hydrochloride	TRZ	Sigma	DMSO	50
	Nialamide	NIA	Sigma	ethanol	10
	Moclobemide	MCL	Sigma	DMSO	10
ANTIPSY. [a]	Haloperidol	HAL	Sigma	DMSO	100
	Chlorpromazine hydrochloride	CPM	Sigma	water	10
	Chlorprotixene hydrochloride	CHX	Sigma	water	10
	Clozapine	CLZ	Sigma	DMSO	10
	Tiapride hydrochloride	TIA	Sigma	water	10
ANTICONV. [b]	Carbamazepine	CBZ	Sigma	DMSO	100
	Lamotrigine	LTG	Richter	DMSO	50
	5,5-diphenyl-hydantoin	DPH	Sigma	DMSO	100
	Topiramate	TOP	HeteroDrugs Limited	DMSO	50
	Zonisamide	ZON	Tocris	water	10
	Gabapentin	GAB	Sigma	water	100
LA / AAR [c]	Bupivacaine hydrochloride	BPV	Sigma	water	10
	Lidocaine hydrochloride monohydrate	LID	Sigma	DMSO	50
	Mexiletine hydrochloride	MEX	Sigma	water	50
	Flecainide acetate	FLC	Tocris	water	10
	Procainamide hydrochloride	PRC	Sigma	water	10
MISCELLANEOUS	Ranolazine dihydrochloride	RAN	Sigma	DMSO	10
	Memantine hydrochloride	MEM	Sigma	water	100
	Riluzole	RIL	Sigma	DMSO	100
	Diclofenac sodium salt	DIC	Sigma	water	50
	Ritanserin	RIT	Tocris	DMSO	50
	Ambroxol hydrochloride	AMB	Tocris	DMSO	50
	Silperisone	SIL	Richter	water	10
	Tolperisone hydrochloride	TOL	Richter	water	10
	Flunarizine dihydrochloride	FLR	Sigma	ethanol	10
	Lifarizine	LIF	Richter	DMSO	10
	Mecamylamine hydrochloride	MEC	Sigma	water	10
	R-(-)-Deprenyl hydrochloride	DPR	Sigma	water	100

Abbr.: [a]antipsychotic agents, [b]anticonvulsants, [c]local anesthetics and antiarrhythmic agents
