# Supplementary material for: Classification of Drugs Based on Properties of Sodium Channel Inhibition: A Comparative Automated Patch-Clamp Study
Source: PLoS One. 2010 Dec 20;5(12):e15568. doi: 10.1371/journal.pone.0015568 (PMC3004914; doi:10.1371/journal.pone.0015568)
Supplement: Materials and Methods S2 — Main electrical properties of cells. (PDF) [file pone.0015568.s002.pdf]

Materials and Methods S2 – Main electrical properties of cells.

Histograms showing the distribution of whole-cell membrane resistance (**A**), series resistance (**B**), whole-cell capacitance values (**C**), and current amplitudes (**D**), evoked by depolarizing voltage steps from -90 to -10 mV. The histograms were constructed from the data of 250 randomly chosen cells. Data from cells having R_{series} > 9 MOhm, or C > 22 pF were excluded from analysis.

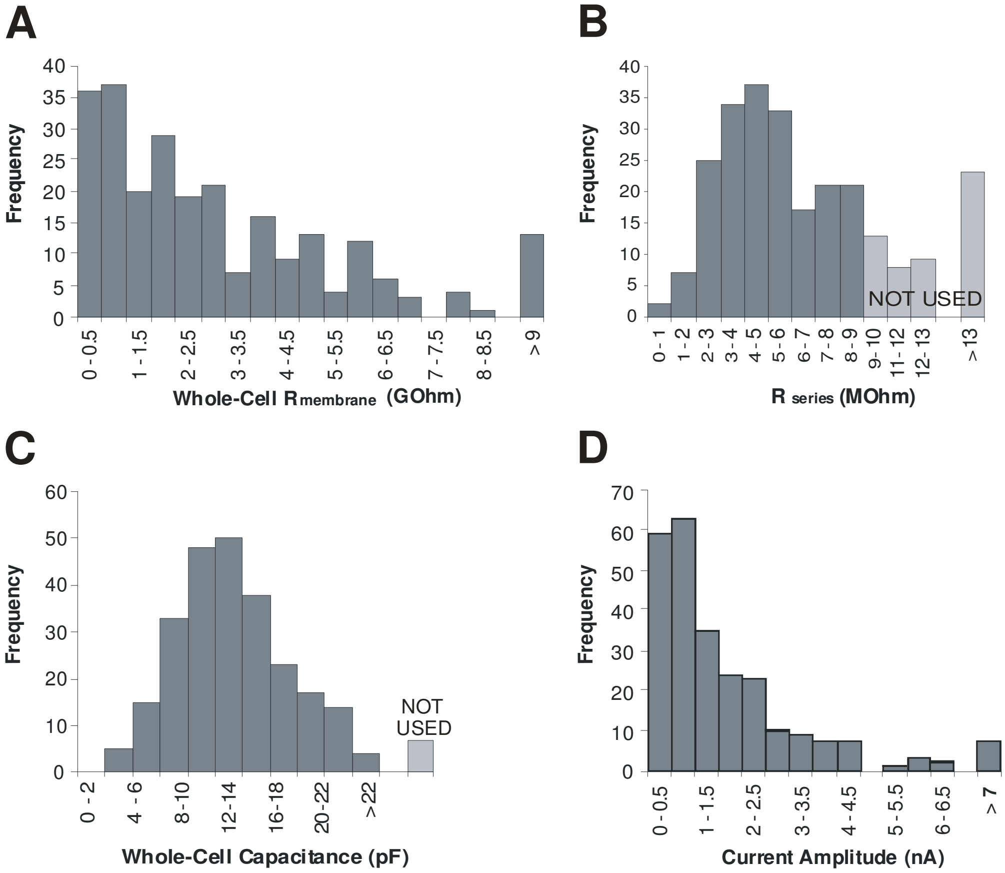

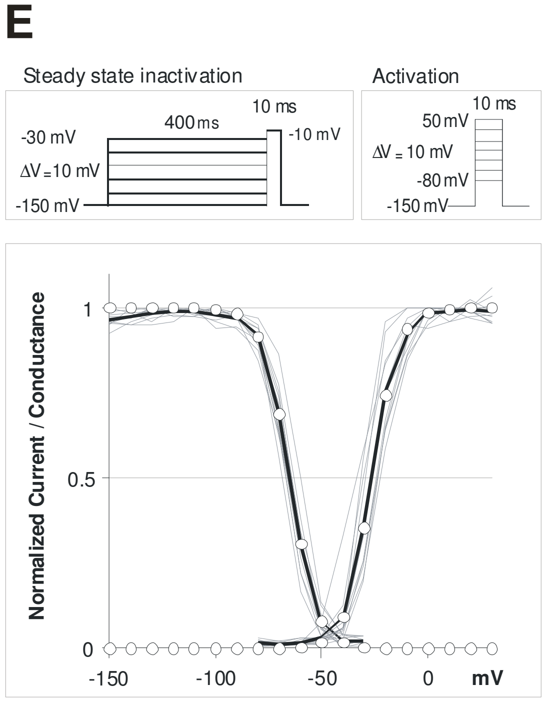

Voltage-conductance curves for activation and inactivation (**E**).

Half-activation and half-inactivation voltages, and slope factors were obtained by fitting the Boltzmann function (open circles) to the average (thick line) of 20 randomly selected curves (thin lines). For activation the values were: $V_{0.5}$ = -26.41 ± 2.74 mV, k = -6.15, for inactivation: $V_{0.5}$ = -65.06 ± 1.56 mV, k = 6.00. Protocols used to test voltage-dependent activation and steady-state inactivation are shown in the upper panels.
