# Supplementary material for: Classification of Drugs Based on Properties of Sodium Channel Inhibition: A Comparative Automated Patch-Clamp Study
Source: PLoS One. 2010 Dec 20;5(12):e15568. doi: 10.1371/journal.pone.0015568 (PMC3004914; doi:10.1371/journal.pone.0015568)

Materials and Methods S3 – Reliability of IC_{50} calculation from single-concentration inhibition values.

Examples for the calculation of IC_{50} values from single concentration measurements

Examples for the effect of 100, 300 and 1000 µM of lidocaine are illustrated on **A, B, C**.

Average inhibition (diamonds in **D**) was 0.09 ± 0.02, 0.23 ± 0.02 and 0.44 ± 0.01 (n = 5 to 7), from which the calculated IC_{50} values are 1048, 977 and 1268 µM, respectively. Right panel illustrates concentration response curves extrapolated from the three single-concentration measurements with the calculated IC_{50} values indicated (red '+' marks).

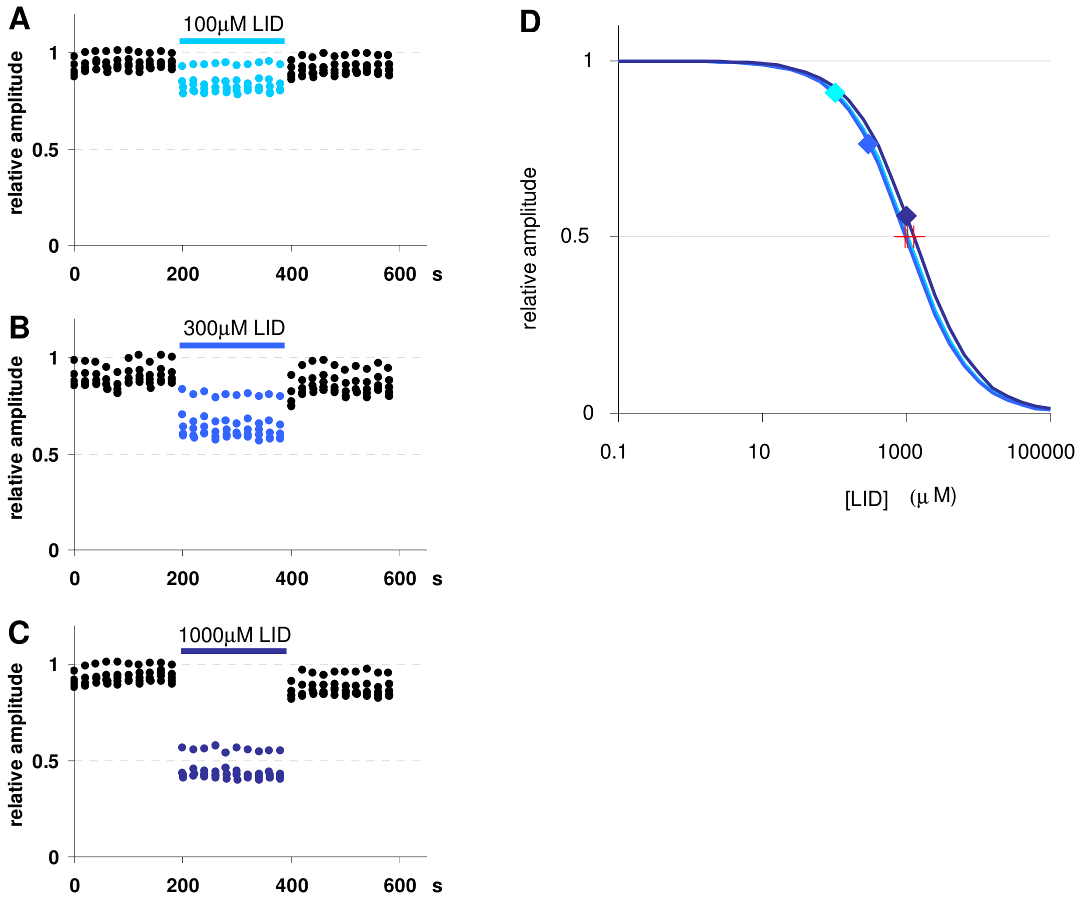

Supplement: Materials and Methods S3 — Reliability of IC50 calculation from single-concentration inhibition values. (PDF) [file pone.0015568.s003.pdf]
