# Supplementary material for: Classification of Drugs Based on Properties of Sodium Channel Inhibition: A Comparative Automated Patch-Clamp Study
Source: PLoS One. 2010 Dec 20;5(12):e15568. doi: 10.1371/journal.pone.0015568 (PMC3004914; doi:10.1371/journal.pone.0015568)
Supplement: Results S1 — Comments on biophysical properties of inhibition. (PDF) [file pone.0015568.s004.pdf]

Results S1 – Comments on biophysical properties of inhibition.

Comment on reversibility

We and others have observed differences in the reversibility measured by the QPatch instrument and by manual patch clamp: washout of lipophilic compounds is delayed in the QPatch. The probable reason for this is the presence of unrecorded cells within the chip. One major difference as compared to manual patch clamp is that QPatch does not allow continuous flow of extracellular medium. It is hypothesized, that because each chip contains about a thousand unrecorded cells attached to the chip surface besides the single recorded one, lipophilic compounds saturate the membrane phase of un-recorded cells, from which the drug may leak even after removal of the drug-containing extracellular solution. Reversibility nevertheless is an additional parameter that can be used for exploration of different types of SCIs, most probably it reflects the lipophilicity of the compounds.

Comment on time constants

If we assumed a single binding reaction, there would be no point in measuring both onset and offset rates. At the IC_{50} value, τ_{on} would be exactly the half of τ_{off} (Onset rate should be concentration-dependent, always faster than offset rate, approaching τ_{off} at low concentrations.) In our measurements, however, onset was often found to be slower than expected based on the extent of inhibition. The fraction of inhibited channels (Inh) can be calculated from τ_{on} and τ_{off} using the formula $(1\text{-Inh}) = \tau_{on} / \tau_{off}$. (The association rate constant contains a concentration term (k_a*cc), while the dissociation rate constant (k_d) does not. The time constants are given by the formulae: $\tau_{on} = 1/(k_a{*}cc + k_d)$ and $\tau_{off} = 1/k_d$. The equilibrium inhibition (Inh) is given by: $(1\text{-Inh}) = k_d/(k_a{*}cc + k_d)$. From these the above formula follows.) In our measurements Inh calculated from the time constants often underestimates experimentally measured Inh values, especially in the case of antidepressants, indicating that processes other than the binding reaction itself determine onset and offset time constants. Because most SCIs are lipophilic, onset and offset kinetics were most likely affected by partitioning into and out of the membrane phase, deprotonation and protonation, translocation within the membrane, diffusion within the membrane, in addition to the actual entry to- and exit from the binding site. Furthermore, binding interactions of different energy levels are supposed to exist within the same binding site, depending on the conformation of the protein and the orientation of the drug molecule [1, 2]. For these reasons, onset time constants were not redundant, they potentially could include additional information which could not be obtained from offset time constants. In addition, τ_{on} could be more accurately measured in the case of drugs with low reversibility, where the measurement τ_{off} was difficult. For these reasons, although τ_{on} was not used for analysis, its correlations with chemical descriptors was calculated.

Comment on use-dependence

As for use-dependence, we point out that its detection is dependent on the voltage protocol used. The absence of detectable use-dependence does not necessarily mean that the drug does not show use-dependent behavior at other stimulation frequencies and holding potential values. Lack of detectable use-dependence may only mean that association/dissociation kinetics of the drug is too fast for the protocol.

References:

[1] Hanck DA, Nikitina E, McNulty MM, Fozzard HA, Lipkind GM, Sheets MF (2009) Using lidocaine and benzocaine to link sodium channel molecular conformations to state-dependent antiarrhythmic drug affinity. Circulation Research 105:492-499.

[2] Kimbrough JT, Gingrich KJ (2000) Quaternary ammonium block of mutant Na+ channels lacking inactivation: features of a transition-intermediate mechanism. The Journal of Physiology 529 Pt 1:93-106.
