# Supplementary material for: Classification of Drugs Based on Properties of Sodium Channel Inhibition: A Comparative Automated Patch-Clamp Study
Source: PLoS One. 2010 Dec 20;5(12):e15568. doi: 10.1371/journal.pone.0015568 (PMC3004914; doi:10.1371/journal.pone.0015568)
Supplement: Results S2 — Detailed discussion of state-dependence. (PDF) [file pone.0015568.s005.pdf]

Results S2 – Detailed discussion of state-dependence.

Since three different measures of potency have been determined, we can compare them by plotting them on the same figure. Each line of the figure represents one individual drug. The slope of the two halves of the lines reflects the two components of state-dependence: K_r/IC_{50} ratio and IC_{50}/K_i ratio. Panel **A)** shows all 35 drugs, while in panel **B)** and **C)** we divided drugs for the sake of clarity: 'Type 1' and 'Type 2' drugs are shown in panel **B)**, while the rest of drugs in panel **C)**.

For data see Table 2 and Table 3.

One would expect that IC_{50} will only be smaller than K_r if the presence of the drug causes channels to accumulate in a non-conducting conformation even at -90 mV holding potential. This requires either slow dissociation or slow inactivated state preference of the drug. On the other hand, for rapidly dissociating drugs, which prefer a depolarized state from which recovery is fast (e.g. fast inactivated state), one would only expect a minor difference between K_r and IC_{50}. (At -90 mV holding potential only a small fraction of the channels is already inactivated – see Materials and Methods S2.)

For the open channel blocker flecainide no difference was found between K_r and IC_{50}. This indicates that it failed to bind to inactivated channels. Ranolazine has also proposed to have preference for open channels [1], and its characteristics were similar to that of flecainide: its $(IC_{50}/K_i) / (K_r/K_i)$ ratio was 0.94.

We observed that for 'Type 1' and 'Type 3' drugs IC_{50} values were typically midway between K_r and K_i, while for many 'Type 2' and 'Type 4' drugs IC_{50} was closer to K_r. Mean $(IC_{50}/K_i) / (K_r/K_i)$ values were 0.55±0.05, 0.83±0.05, 0.48±0.01, and 0.79±0.05, for 'Type 1', 'Type 2', 'Type 3' and 'Type 4' drugs, respectively.

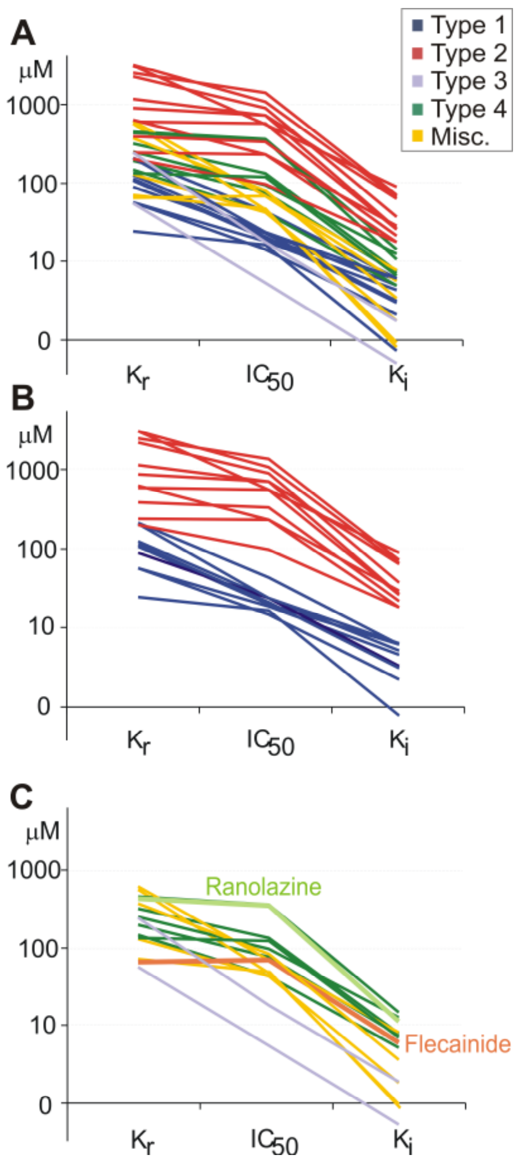

Reference:

[1] Wang GK, Calderon J, Wang SY (2008) State- and use-dependent block of muscle Nav1.4 and neuronal Nav1.7 voltage-gated Na+ channel isoforms by ranolazine. Mol Pharmacol 73, 940-948.
