# Supplementary material for: Classification of Drugs Based on Properties of Sodium Channel Inhibition: A Comparative Automated Patch-Clamp Study
Source: PLoS One. 2010 Dec 20;5(12):e15568. doi: 10.1371/journal.pone.0015568 (PMC3004914; doi:10.1371/journal.pone.0015568)
Supplement: Results S3 — Cross-correlations of 58 chemical descriptors, based on the chemical properties of the 44 drugs we used. (PDF) [file pone.0015568.s006.pdf]

CROSS-CORRELATIONS OF 58 CHEMICAL DESCRIPTORS, BASED ON THE CHEMICAL PROPERTIES OF THE 44 DRUGS WE USED

For the method of calculation of chemical descriptors see www.chemaxon.com.

	AcceptorCount	AcceptorSiteCount	AliphaticAtomCount	AliphaticBondCount	AliphaticRingCount	AromaticAtomCount	AromaticBondCount	AromaticRingCount	AsymmetricAtomCount	AtomCount	BalabanIndex	BasicpKa	BondCount	CarboAromaticRingCount	CarboRingCount	ChainAtomCount	ChainBondCount	ChiralCenterCount	DominantTautomerCount	DonorCount	DonorSiteCount	DreidingEnergy	ExactMass	FusedRingCount	HararyIndex	HeteroRingCount	HyperWienerIndex	LargestRingSize	LargestRingSystemSize	Length perp.to max area	Length perp.to min area	log N(pKa)	logD 6	logD 7.3	logD 8.6	logP	Max-proj-area	Max-proj-radius	MicrospeciesCount	Min-proj-area	Min-proj-radius	MolecularPolarizability	PSA 7.3	RandicIndex	Refractivity	RingAtomCount	RingBondCount	RingCount	RingSystemCount	RotatableBondCount	SmallestRingSize	SmallestRingSystemSize	StereoIsomerCount	SzegedIndex	TautomerCount	Tetrahedr.Stereoisom.Cour	van der Waals volume	WienerIndex
AcceptorCount		0.96	0.50	0.48	-0.04	0.16	0.17	0.19	0.07	0.24	-0.27	-0.20	0.24	0.01	-0.30	0.46	0.54	-0.02	0.19	0.31	0.30	0.16	0.63	-0.17	0.47	0.42	0.46	-0.12	-0.20	0.04	0.43	0.23	0.13	0.07	-0.02	-0.12	0.47	0.40	0.63	0.23	0.39	0.30	0.68	0.46	0.27	0.23	0.18	0.14	0.35	0.43	-0.25	-0.27	0.12	0.44	0.12	0.12	0.39	0.48
AcceptorSiteCount	0.96		0.54	0.50	-0.07	0.11	0.13	0.15	0.07	0.23	-0.21	-0.35	0.22	-0.04	-0.38	0.53	0.60	-0.05	0.28	0.39	0.36	0.20	0.60	-0.19	0.45	0.41	0.45	-0.11	-0.22	0.06	0.41	0.40	0.08	0.00	-0.15	-0.29	0.43	0.39	0.64	0.19	0.34	0.28	0.80	0.44	0.24	0.17	0.12	0.08	0.32	0.47	-0.28	-0.27	0.13	0.42	0.26	0.14	0.34	0.47
AliphaticAtomCount	0.50	0.54		0.97	0.41	-0.23	-0.24	-0.24	0.42	0.62	-0.14	0.18	0.59	-0.19	-0.14	0.63	0.67	0.38	-0.15	0.13	0.00	0.32	0.53	-0.15	0.47	0.23	0.48	-0.17	-0.14	0.27	0.42	0.13	-0.22	-0.14	-0.12	-0.17	0.50	0.40	0.66	0.48	0.54	0.40	0.30	0.45	0.34	0.13	0.09	0.10	0.25	0.56	-0.07	-0.14	0.44	0.44	-0.05	0.44	0.55	0.50
AliphaticBondCount	0.48	0.50	0.97		0.54	-0.05	-0.06	-0.07	0.39	0.73	-0.33	0.19	0.72	-0.03	0.02	0.49	0.57	0.37	-0.18	0.04	-0.10	0.48	0.64	-0.02	0.63	0.37	0.58	-0.07	-0.01	0.37	0.47	0.06	-0.08	0.00	0.02	-0.03	0.61	0.47	0.65	0.61	0.65	0.56	0.23	0.60	0.50	0.35	0.33	0.33	0.36	0.52	-0.12	-0.04	0.43	0.58	-0.09	0.43	0.67	0.61
AliphaticRingCount	-0.04	-0.07	0.41	0.54		-0.17	-0.17	-0.19	0.51	0.25	-0.32	0.16	0.29	-0.13	0.28	-0.37	-0.35	0.62	-0.27	-0.22	-0.21	0.32	0.11	0.60	0.22	0.42	0.03	0.16	0.61	0.07	-0.14	-0.17	-0.09	-0.07	-0.03	0.05	0.05	-0.14	0.24	0.27	0.30	0.16	-0.10	0.13	0.15	0.36	0.44	0.57	0.02	-0.34	-0.17	0.51	0.62	0.13	-0.30	0.62	0.16	0.06
AromaticAtomCount	0.16	0.11	-0.23	-0.05	-0.17		1.00	0.99	-0.49	0.52	-0.74	-0.07	0.56	0.87	0.46	-0.16	0.04	-0.52	0.05	-0.24	-0.33	0.64	0.65	0.10	0.74	0.43	0.64	0.29	0.10	0.47	0.57	-0.10	0.74	0.77	0.71	0.61	0.64	0.61	-0.06	0.53	0.45	0.75	-0.10	0.76	0.78	0.83	0.80	0.71	0.64	0.29	-0.20	-0.01	-0.49	0.73	0.04	-0.51	0.61	0.68
AromaticBondCount	0.17	0.13	-0.24	-0.06	-0.17	1.00		0.99	-0.49	0.51	-0.74	-0.09	0.55	0.85	0.44	-0.16	0.04	-0.53	0.05	-0.25	-0.33	0.65	0.65	0.11	0.73	0.45	0.64	0.27	0.10	0.46	0.57	-0.08	0.75	0.77	0.71	0.60	0.63	0.60	-0.06	0.52	0.44	0.74	-0.08	0.76	0.77	0.82	0.80	0.71	0.64	0.28	-0.23	-0.02	-0.49	0.72	0.04	-0.51	0.60	0.67
AromaticRingCount	0.19	0.15	-0.24	-0.07	-0.19	0.99	0.99		-0.50	0.49	-0.74	-0.11	0.53	0.81	0.41	-0.16	0.03	-0.53	0.04	-0.26	-0.33	0.65	0.64	0.11	0.72	0.48	0.65	0.24	0.09	0.45	0.57	-0.05	0.75	0.77	0.70	0.58	0.62	0.61	-0.05	0.50	0.42	0.72	-0.04	0.74	0.75	0.81	0.79	0.71	0.64	0.27	-0.30	-0.03	-0.49	0.73	0.04	-0.50	0.58	0.68
AsymmetricAtomCount	0.07	0.07	0.42	0.39	0.51	-0.49	-0.49	-0.50		-0.01	0.22	0.07	-0.02	-0.42	-0.13	0.08	0.02	0.87	-0.17	-0.01	0.00	-0.05	-0.14	0.17	-0.11	0.05	-0.16	-0.25	0.15	0.00	-0.30	-0.02	-0.35	-0.35	-0.29	-0.21	-0.21	-0.30	0.37	0.00	-0.02	-0.18	0.10	-0.18	-0.25	-0.19	-0.16	-0.05	-0.20	-0.14	-0.19	0.13	0.88	-0.16	-0.13	0.89	-0.09	-0.15
AtomCount	0.24	0.23	0.62	0.73	0.25	0.52	0.51	0.49	-0.01		-0.64	0.31	1.00	0.49	0.34	0.34	0.52	-0.05	-0.23	-0.21	-0.40	0.76	0.82	-0.05	0.89	0.40	0.85	0.08	-0.04	0.73	0.77	-0.08	0.29	0.45	0.49	0.39	0.88	0.78	0.39	0.88	0.75	0.93	-0.08	0.90	0.89	0.70	0.66	0.59	0.66	0.67	-0.11	-0.09	-0.03	0.88	-0.09	-0.04	0.95	0.88
BalabanIndex	-0.27	-0.21	-0.14	-0.33	-0.32	-0.74	-0.74	-0.74	0.22	-0.64		-0.07	-0.68	-0.60	-0.45	0.18	-0.01	0.15	0.01	0.26	0.36	-0.68	-0.72	-0.29	-0.77	-0.61	-0.65	-0.22	-0.28	-0.44	-0.66	0.13	-0.56	-0.59	-0.56	-0.51	-0.72	-0.67	-0.17	-0.57	-0.50	-0.77	0.07	-0.78	-0.78	-0.87	-0.87	-0.85	-0.63	-0.25	0.23	-0.14	0.14	-0.74	-0.02	0.15	-0.70	-0.69
BasicpKa	-0.20	-0.35	0.18	0.19	0.16	-0.07	-0.09	-0.11	0.07	0.31	-0.07		0.29	0.01	0.28	0.09	0.07	0.16	-0.53	-0.28	-0.33	0.03	0.04	0.08	0.06	-0.21	0.06	0.01	0.09	0.12	0.13	-0.49	-0.28	-0.06	0.22	0.31	0.20	0.13	-0.22	0.25	0.21	0.17	-0.55	0.07	0.19	0.01	0.02	0.02	-0.07	0.16	0.37	0.03	-0.08	0.10	-0.35	-0.09	0.25	0.07
BondCount	0.24	0.22	0.59	0.72	0.29	0.56	0.55	0.53	-0.02	1.00	-0.68	0.29		0.52	0.37	0.28	0.47	-0.05	-0.23	-0.24	-0.42	0.79	0.83	0.00	0.91	0.45	0.86	0.11	0.02	0.73	0.76	-0.09	0.33	0.48	0.51	0.42	0.88	0.77	0.38	0.88	0.76	0.94	-0.09	0.91	0.91	0.75	0.71	0.65	0.67	0.63	-0.14	-0.05	-0.03	0.89	-0.10	-0.04	0.95	0.89
CarboAromaticRingCount	0.01	-0.04	-0.19	-0.03	-0.13	0.87	0.85	0.81	-0.42	0.49	-0.60	0.01	0.52		0.62	-0.11	0.05	-0.44	-0.11	-0.22	-0.36	0.54	0.56	0.10	0.64	0.15	0.48	0.36	0.11	0.40	0.43	-0.29	0.62	0.66	0.67	0.66	0.55	0.46	-0.13	0.51	0.49	0.69	-0.27	0.67	0.70	0.70	0.68	0.59	0.50	0.29	0.04	0.04	-0.46	0.58	-0.12	-0.48	0.53	0.53
CarboRingCount	-0.30	-0.38	-0.14	0.02	0.28	0.46	0.44	0.41	-0.13	0.34	-0.45	0.28	0.37	0.62		-0.29	-0.20	0.11	-0.28	-0.12	-0.22	0.36	0.25	0.30	0.36	-0.22	0.24	0.13	0.31	0.30	0.07	-0.52	0.30	0.30	0.38	0.56	0.20	0.11	-0.28	0.41	0.32	0.43	-0.44	0.34	0.45	0.48	0.51	0.55	0.27	0.00	0.09	0.26	-0.11	0.34	-0.24	-0.13	0.31	0.26
ChainAtomCount	0.46	0.53	0.63	0.49	-0.37	-0.16	-0.16	-0.16	0.08	0.34	0.18	0.09	0.28	-0.11	-0.29		0.95	-0.01	0.02	0.30	0.17	0.05	0.35	-0.43	0.23	-0.22	0.34	-0.23	-0.42	0.17	0.41	0.21	-0.22	-0.19	-0.18	-0.24	0.37	0.39	0.45	0.21	0.27	0.20	0.37	0.26	0.16	-0.29	-0.35	-0.40	-0.01	0.80	0.17	-0.30	0.03	0.24	0.23	0.03	0.32	0.34
ChainBondCount	0.54	0.60	0.67	0.57	-0.35	0.04	0.04	0.03	0.02	0.52	-0.01	0.07	0.47	0.05	-0.20	0.95		-0.08	0.06	0.28	0.12	0.19	0.54	-0.54	0.43	-0.09	0.56	-0.30	-0.53	0.33	0.59	0.20	-0.06	-0.01	-0.02	-0.11	0.56	0.59	0.49	0.38	0.39	0.40	0.37	0.47	0.36	-0.06	-0.14	-0.22	0.28	0.90	0.05	-0.45	-0.03	0.47	0.22	-0.03	0.53	0.56
ChiralCenterCount	-0.02	-0.05	0.38	0.37	0.62	-0.52	-0.53	-0.53	0.87	-0.05	0.15	0.16	-0.05	-0.44	0.11	-0.01	-0.08		-0.22	0.10	0.14	-0.13	-0.15	0.24	-0.16	-0.09	-0.21	-0.27	0.24	-0.06	-0.32	-0.17	-0.40	-0.42	-0.33	-0.19	-0.25	-0.32	0.26	-0.04	-0.04	-0.21	0.05	-0.23	-0.26	-0.20	-0.15	0.00	-0.23	-0.22	-0.09	0.24	0.85	-0.20	-0.17	0.86	-0.13	-0.21
DominantTautomerCount	0.19	0.28	-0.15	-0.18	-0.27	0.05	0.05	0.04	-0.17	-0.23	0.01	-0.53	-0.23	-0.11	-0.28	0.02	0.06	-0.22		0.51	0.53	-0.16	-0.07	-0.32	-0.09	0.06	-0.04	-0.15	-0.31	-0.02	0.02	0.44	0.07	-0.04	-0.21	-0.36	-0.09	0.03	0.16	-0.17	-0.22	-0.16	0.47	-0.06	-0.11	-0.07	-0.12	-0.16	0.14	-0.01	-0.08	-0.23	-0.09	-0.09	0.74	-0.09	-0.16	-0.05
DonorCount	0.31	0.39	0.13	0.04	-0.22	-0.24	-0.25	-0.26	-0.01	-0.21	0.26	-0.28	-0.24	-0.22	-0.12	0.30	0.28	0.10	0.51		0.91	-0.36	0.00	-0.38	-0.16	-0.34	-0.09	-0.19	-0.37	-0.13	-0.07	0.24	-0.40	-0.53	-0.62	-0.58	-0.13	-0.08	0.17	-0.19	-0.13	-0.22	0.61	-0.14	-0.21	-0.31	-0.35	-0.38	0.00	0.16	0.12	-0.31	0.00	-0.16	0.53	0.02	-0.19	-0.10
DonorSiteCount	0.30	0.36	0.00	-0.10	-0.21	-0.33	-0.33	-0.33	0.00	-0.40	0.36	-0.33	-0.42	-0.36	-0.22	0.17	0.12	0.14	0.53	0.91		-0.47	-0.16	-0.30	-0.32	-0.31	-0.21	-0.18	-0.30	-0.30	-0.21	0.31	-0.39	-0.54	-0.65	-0.65	-0.32	-0.24	0.18	-0.39	-0.31	-0.39	0.68	-0.32	-0.38	-0.40	-0.43	-0.43	-0.12	-0.06	0.06	-0.22	0.09	-0.28	0.42	0.11	-0.39	-0.24
DreidingEnergy	0.16	0.20	0.32	0.48	0.32	0.64	0.65	0.65	-0.05	0.76	-0.68	0.03	0.79	0.54	0.36	0.05	0.19	-0.13	-0.16	-0.36	-0.47		0.70	0.36	0.84	0.60	0.72	0.25	0.35	0.49	0.55	0.11	0.48	0.54	0.49	0.36	0.69	0.58	0.26	0.63	0.61	0.83	0.03	0.80	0.83	0.77	0.79	0.78	0.48	0.29	-0.30	0.25	-0.01	0.80	-0.04	-0.03	0.71	0.76
ExactMass	0.63	0.60	0.53	0.64	0.11	0.65	0.65	0.64	-0.14	0.82	-0.72	0.04	0.83	0.56	0.25	0.35	0.54	-0.15	-0.07	0.00	-0.16	0.70		-0.05	0.94	0.51	0.87	0.07	-0.06	0.52	0.80	0.00	0.47	0.53	0.49	0.37	0.90	0.80	0.44	0.74	0.75	0.91	0.25	0.94	0.88	0.75	0.70	0.62	0.72	0.63	-0.22	-0.16	-0.09	0.91	0.00	-0.11	0.89	0.91
FusedRingCount	-0.17	-0.19	-0.15	-0.02	0.60	0.10	0.11	0.11	0.17	-0.05	-0.29	0.08	0.00	0.10	0.30	-0.43	-0.54	0.24	-0.32	-0.38	-0.30	0.36	-0.05		0.07	0.35	-0.20	0.55	0.99	-0.12	-0.30	-0.16	0.07	0.04	0.07	0.13	-0.12	-0.31	-0.09	0.02	0.14	0.04	-0.12	0.00	0.07	0.24	0.39	0.53	-0.40	-0.51	-0.08	0.88	0.26	-0.06	-0.21	0.26	-0.12	-0.15
HararyIndex	0.47	0.45	0.47	0.63	0.22	0.74	0.73	0.72	-0.11	0.89	-0.77	0.06	0.91	0.64	0.36	0.23	0.43	-0.16	-0.09	-0.16	-0.32	0.84	0.94	0.07		0.59	0.90	0.17	0.07	0.61	0.74	-0.01	0.53	0.60	0.56	0.44	0.89	0.77	0.39	0.82	0.79	0.95	0.11	0.99	0.94	0.86	0.83	0.76	0.73	0.57	-0.27	-0.04	-0.09	0.96	-0.04	-0.10	0.91	0.94
HeteroRingCount	0.42	0.41	0.23	0.37	0.42	0.43	0.45	0.48	0.05	0.40	-0.61	-0.21	0.45	0.15	-0.22	-0.22	-0.09	-0.09	0.06	-0.34	-0.31	0.60	0.51	0.35	0.59		0.46	0.26	0.33	0.24	0.38	0.26	0.40	0.44	0.33	0.13	0.48	0.38	0.39	0.36	0.38	0.48	0.24	0.55	0.47	0.68	0.70	0.70	0.41	-0.02	-0.51	0.17	0.14	0.53	-0.01	0.14	0.45	0.49
HyperWienerIndex	0.46	0.45	0.48	0.58	0.03	0.64	0.64	0.65	-0.16	0.85	-0.65	0.06	0.86	0.48	0.24	0.34	0.56	-0.21	-0.04	-0.09	-0.21	0.72	0.87	-0.20	0.90	0.46		-0.07	-0.21	0.62	0.82	0.12	0.47	0.53	0.46	0.31	0.86	0.85	0.46	0.72	0.61	0.87	0.18	0.91	0.84	0.71	0.64	0.57	0.79	0.68	-0.33	-0.27	-0.15	0.97	0.03	-0.16	0.88	0.99
LargestRingSize	-0.12	-0.11	-0.17	-0.07	0.16	0.29	0.27	0.24	-0.25	0.08	-0.22	0.01	0.11	0.36	0.13	-0.23	-0.30	-0.27	-0.15	-0.19	-0.18	0.25	0.07	0.55	0.17	0.26	-0.07		0.60	-0.04	-0.04	-0.06	0.13	0.16	0.17	0.10	0.05	-0.09	-0.12	0.10	0.22	0.17	-0.14	0.16	0.20	0.27	0.36	0.32	-0.24	-0.21	0.33	0.54	-0.25	0.02	-0.06	-0.25	-0.01	-0.02
LargestRingSystemSize	-0.20	-0.22	-0.14	-0.01	0.61	0.10	0.10	0.09	0.15	-0.04	-0.28	0.09	0.02	0.11	0.31	-0.42	-0.53	0.24	-0.31	-0.37	-0.30	0.35	-0.06	0.99	0.07	0.33	-0.21	0.60		-0.11	-0.29	-0.17	0.06	0.04	0.07	0.14	-0.11	-0.30	-0.09	0.02	0.16	0.04	-0.16	0.00	0.08	0.23	0.39	0.51	-0.42	-0.49	0.01	0.92	0.24	-0.07	-0.20	0.23	-0.11	-0.16
Length perp.to max area	0.04	0.06	0.27	0.37	0.07	0.47	0.46	0.45	0.00	0.73	-0.44	0.12	0.73	0.40	0.30	0.17	0.33	-0.06	-0.02	-0.13	-0.30	0.49	0.52	-0.12	0.61	0.24	0.62	-0.04	-0.11		0.55	-0.01	0.26	0.34	0.34	0.28	0.56	0.55	0.22	0.81	0.43	0.70	-0.05	0.62	0.66	0.51	0.47	0.42	0.56	0.51	-0.21	-0.13	-0.01	0.62	0.07	0.00	0.74	0.62
Length perp.to min area	0.43	0.41	0.42	0.47	-0.14	0.57	0.57	0.57	-0.30	0.77	-0.66	0.13	0.76	0.43	0.07	0.41	0.59	-0.32	0.02	-0.07	-0.21	0.55	0.80	-0.30	0.74	0.38	0.82	-0.04	-0.29	0.55		0.15	0.36	0.46	0.45	0.28	0.89	0.99	0.39	0.58	0.44	0.79	0.11	0.80	0.78	0.56	0.48	0.38	0.67	0.74	-0.12	-0.34	-0.23	0.80	0.13	-0.25	0.84	0.83
log N(pKa)	0.23	0.40	0.13	0.06	-0.17	-0.10	-0.08	-0.05	-0.02	-0.08	0.13	-0.49	-0.09	-0.29	-0.52	0.21	0.20	-0.17	0.44	0.24	0.31	0.11	0.00	-0.16	-0.01	0.26	0.12	-0.06	-0.17	-0.01	0.15		0.01	-0.06	-0.31	-0.66	-0.06	0.15	0.28	-0.22	-0.28	-0.09	0.62	-0.01	-0.11	-0.12	-0.14	-0.16	-0.02	0.10	-0.28	-0.07	0.06	0.04	0.47	0.07	-0.11	0.09
logD 6	0.13	0.08	-0.22	-0.08	-0.09	0.74	0.75	0.75	-0.35	0.29	-0.56	-0.28	0.33	0.62	0.30	-0.22	-0.06	-0.40	0.07	-0.40	-0.39	0.48	0.47	0.07	0.53	0.40	0.47	0.13	0.06	0.26	0.36	0.01		0.94	0.79	0.67	0.38	0.38	-0.02	0.32	0.27	0.51	-0.12	0.53	0.50	0.64	0.62	0.57	0.53	0.06	-0.28	0.05	-0.27	0.53	-0.07	-0.30	0.38	0.48
logD 7.3	0.07	0.00	-0.14	0.00	-0.07	0.77	0.77	0.77	-0.35	0.45	-0.59	-0.06	0.48	0.66	0.30	-0.19	-0.01	-0.42	-0.04	-0.53	-0.54	0.54	0.53	0.04	0.60	0.44	0.53	0.16	0.04	0.34	0.46	-0.06	0.94		0.94	0.76	0.51	0.49	-0.03	0.45	0.38	0.62	-0.28	0.61	0.61	0.70	0.67	0.59	0.57	0.13	-0.21	0.00	-0.33	0.60	-0.17	-0.35	0.52	0.56
logD 8.6	-0.02	-0.15	-0.12	0.02	-0.03	0.71	0.71	0.70	-0.29	0.49	-0.56	0.22	0.51	0.67	0.38	-0.18	-0.02	-0.33	-0.21	-0.62	-0.65	0.49	0.49	0.07	0.56	0.33	0.46	0.17	0.07	0.34	0.45	-0.31	0.79	0.94		0.88	0.53	0.47	-0.12	0.50	0.45	0.62	-0.48	0.57	0.62	0.66	0.64	0.56	0.50	0.14	-0.08	0.02	-0.32	0.55	-0.30	-0.35	0.55	0.50
logP	-0.12	-0.29	-0.17	-0.03	0.05	0.61	0.60	0.58	-0.21	0.39	-0.51	0.31	0.42	0.66	0.56	-0.24	-0.11	-0.19	-0.36	-0.58	-0.65	0.36	0.37	0.13	0.44	0.13	0.31	0.10	0.14	0.28	0.28	-0.66	0.67	0.76	0.88		0.42	0.31	-0.25	0.47	0.42	0.51	-0.64	0.44	0.52	0.57	0.56	0.52	0.40	0.06	0.01	0.06	-0.24	0.41	-0.42	-0.27	0.47	0.35
Max-proj-area	0.47	0.43	0.50	0.61	0.05	0.64	0.63	0.62	-0.21	0.88	-0.72	0.20	0.88	0.55	0.20	0.37	0.56	-0.25	-0.09	-0.13	-0.32	0.69	0.90	-0.12	0.89	0.48	0.86	0.05	-0.11	0.56	0.89	-0.06	0.38	0.51	0.53	0.42		0.90	0.42	0.76	0.77	0.91	0.04	0.92	0.91	0.71	0.65	0.56	0.69	0.70	-0.11	-0.20	-0.21	0.89	0.01	-0.23	0.95	0.89
Max-proj-radius	0.40	0.39	0.40	0.47	-0.14	0.61	0.60	0.61	-0.30	0.78	-0.67	0.13	0.77	0.46	0.11	0.39	0.59	-0.32	0.03	-0.08	-0.24	0.58	0.80	-0.31	0.77	0.38	0.85	-0.09	-0.30	0.55	0.99	0.15	0.38	0.49	0.47	0.31	0.90		0.36	0.59	0.46	0.80	0.08	0.83	0.80	0.59	0.51	0.41	0.70	0.75	-0.16	-0.35	-0.26	0.83	0.13	-0.27	0.85	0.85
MicrospeciesCount	0.63	0.64	0.66	0.65	0.24	-0.06	-0.06	-0.05	0.37	0.39	-0.17	-0.22	0.38	-0.13	-0.28	0.45	0.49	0.26	0.16	0.17	0.18	0.26	0.44	-0.09	0.39	0.39	0.46	-0.12	-0.09	0.22	0.39	0.28	-0.02	-0.03	-0.12	-0.25	0.42	0.36		0.27	0.32	0.29	0.54	0.38	0.24	0.15	0.13	0.13	0.23	0.38	-0.25	-0.08	0.45	0.39	0.05	0.46	0.41	0.44
Min-proj-area	0.23	0.19	0.48	0.61	0.27	0.53	0.52	0.50	0.00	0.88	-0.57	0.25	0.88	0.51	0.41	0.21	0.38	-0.04	-0.17	-0.19	-0.39	0.63	0.74	0.02	0.82	0.36	0.72	0.10	0.02	0.81	0.58	-0.22	0.32	0.45	0.50	0.47	0.76	0.59	0.27		0.82	0.84	-0.10	0.81	0.83	0.69	0.66	0.61	0.62	0.54	-0.19	-0.06	-0.01	0.77	-0.07	-0.01	0.89	0.76
Min-proj-radius	0.39	0.34	0.54	0.65	0.30	0.45	0.44	0.42	-0.02	0.75	-0.50	0.21	0.76	0.49	0.32	0.27	0.39	-0.04	-0.22	-0.13	-0.31	0.61	0.75	0.14	0.79	0.38	0.61	0.22	0.16	0.43	0.44	-0.28	0.27	0.38	0.45	0.42	0.77	0.46	0.32	0.82		0.75	-0.02	0.77	0.75	0.62	0.61	0.57	0.46	0.45	-0.07	0.07	-0.05	0.69	-0.18	-0.07	0.77	0.67
MolecularPolarizability	0.30	0.28	0.40	0.56	0.16	0.75	0.74	0.72	-0.18	0.93	-0.77	0.17	0.94	0.69	0.43	0.20	0.40	-0.21	-0.16	-0.22	-0.39	0.83	0.91	0.04	0.95	0.48	0.87	0.17	0.04	0.70	0.79	-0.09	0.51	0.62	0.62	0.51	0.91	0.80	0.29	0.84	0.75		-0.03	0.96	0.98	0.84	0.80	0.72	0.72	0.58	-0.15	-0.05	-0.16	0.93	-0.06	-0.18	0.94	0.91
PSA 7.3	0.68	0.80	0.30	0.23	-0.10	-0.10	-0.08	-0.04	0.10	-0.08	0.07	-0.55	-0.09	-0.27	-0.44	0.37	0.37	0.05	0.47	0.61	0.68	0.03	0.25	-0.12	0.11	0.24	0.18	-0.14	-0.16	-0.05	0.11	0.62	-0.12	-0.28	-0.48	-0.64	0.04	0.08	0.54	-0.10	-0.02	-0.03		0.08	-0.06	-0.10	-0.12	-0.11	0.07	0.19	-0.35	-0.14	0.23	0.11	0.44	0.25	-0.03	0.17
RandicIndex	0.46	0.44	0.45	0.60	0.13	0.76	0.76	0.74	-0.18	0.90	-0.78	0.07	0.91	0.67	0.34	0.26	0.47	-0.23	-0.06	-0.14	-0.32	0.80	0.94	0.00	0.99	0.55	0.91	0.16	0.00	0.62	0.80	-0.01	0.53	0.61	0.57	0.44	0.92	0.83	0.38	0.81	0.77	0.96	0.08		0.95	0.85	0.80	0.72	0.76	0.63	-0.22	-0.11	-0.17	0.96	0.00	-0.18	0.93	0.95
Refractivity	0.27	0.24	0.34	0.50	0.15	0.78	0.77	0.75	-0.25	0.89	-0.78	0.19	0.91	0.70	0.45	0.16	0.36	-0.26	-0.11	-0.21	-0.38	0.83	0.88	0.07	0.94	0.47	0.84	0.20	0.08	0.66	0.78	-0.11	0.50	0.61	0.62	0.52	0.91	0.80	0.24	0.83	0.75	0.98	-0.06	0.95		0.84	0.81	0.74	0.69	0.53	-0.11	-0.05	-0.22	0.91	-0.01	-0.24	0.91	0.88
RingAtomCount	0.23	0.17	0.13	0.35	0.36	0.83	0.82	0.81	-0.19	0.70	-0.87	0.01	0.75	0.70	0.48	-0.29	-0.06	-0.20	-0.07	-0.31	-0.40	0.77	0.75	0.24	0.86	0.68	0.71	0.27	0.23	0.51	0.56	-0.12	0.64	0.70	0.66	0.57	0.71	0.59	0.15	0.69	0.62	0.84	-0.10	0.85	0.84		0.99	0.94	0.76	0.18	-0.33	0.06	-0.15	0.82	-0.13	-0.16	0.74	0.76
RingBondCount	0.18	0.12	0.09	0.33	0.44	0.80	0.80	0.79	-0.16	0.66	-0.87	0.02	0.71	0.68	0.51	-0.35	-0.14	-0.15	-0.12	-0.35	-0.43	0.79	0.70	0.39	0.83	0.70	0.64	0.36	0.39	0.47	0.48	-0.14	0.62	0.67	0.64	0.56	0.65	0.51	0.13	0.66	0.61	0.80	-0.12	0.80	0.81	0.99		0.98	0.65	0.08	-0.31	0.21	-0.10	0.77	-0.16	-0.11	0.68	0.69
RingCount	0.14	0.08	0.10	0.33	0.57	0.71	0.71	0.71	-0.05	0.59	-0.85	0.02	0.65	0.59	0.55	-0.40	-0.22	0.00	-0.16	-0.38	-0.43	0.78	0.62	0.53	0.76	0.70	0.57	0.32	0.51	0.42	0.38	-0.16	0.57	0.59	0.56	0.52	0.56	0.41	0.13	0.61	0.57	0.72	-0.11	0.72	0.74	0.94	0.98		0.55	-0.02	-0.38	0.34	0.04	0.70	-0.18	0.02	0.61	0.61
RingSystemCount	0.35	0.32	0.25	0.36	0.02	0.64	0.64	0.64	-0.20	0.66	-0.63	-0.07	0.67	0.50	0.27	-0.01	0.28	-0.23	0.14	0.00	-0.12	0.48	0.72	-0.40	0.73	0.41	0.79	-0.24	-0.42	0.56	0.67	-0.02	0.53	0.57	0.50	0.40	0.69	0.70	0.23	0.62	0.46	0.72	0.07	0.76	0.69	0.76	0.65	0.55		0.46	-0.40	-0.52	-0.19	0.80	0.01	-0.20	0.74	0.79
RotatableBondCount	0.43	0.47	0.56	0.52	-0.34	0.29	0.28	0.27	-0.14	0.67	-0.25	0.16	0.63	0.29	0.00	0.80	0.90	-0.22	-0.01	0.16	-0.06	0.29	0.63	-0.51	0.57	-0.02	0.68	-0.21	-0.49	0.51	0.74	0.10	0.06	0.13	0.14	0.06	0.70	0.75	0.38	0.54	0.45	0.58	0.19	0.63	0.53	0.18	0.08	-0.02	0.46		0.02	-0.43	-0.22	0.60	0.18	-0.22	0.69	0.68
SmallestRingSize	-0.25	-0.28	-0.07	-0.12	-0.17	-0.20	-0.23	-0.30	-0.19	-0.11	0.23	0.37	-0.14	0.04	0.09	0.17	0.05	-0.09	-0.08	0.12	0.06	-0.30	-0.22	-0.08	-0.27	-0.51	-0.33	0.33	0.01	-0.21	-0.12	-0.28	-0.28	-0.21	-0.08	0.01	-0.11	-0.16	-0.25	-0.19	-0.07	-0.15	-0.35	-0.22	-0.11	-0.33	-0.31	-0.38	-0.40	0.02		0.10	-0.28	-0.31	0.04	-0.28	-0.17	-0.31
SmallestRingSystemSize	-0.27	-0.27	-0.14	-0.04	0.51	-0.01	-0.02	-0.03	0.13	-0.09	-0.14	0.03	-0.05	0.04	0.26	-0.30	-0.45	0.24	-0.23	-0.31	-0.22	0.25	-0.16	0.88	-0.04	0.17	-0.27	0.54	0.92	-0.13	-0.34	-0.07	0.05	0.00	0.02	0.06	-0.20	-0.35	-0.08	-0.06	0.07	-0.05	-0.14	-0.11	-0.05	0.06	0.21	0.34	-0.52	-0.43	0.10		0.25	-0.16	-0.14	0.24	-0.19	-0.23
StereoIsomerCount	0.12	0.13	0.44	0.43	0.62	-0.49	-0.49	-0.49	0.88	-0.03	0.14	-0.08	-0.03	-0.46	-0.11	0.03	-0.03	0.85	-0.09	0.00	0.09	-0.01	-0.09	0.26	-0.09	0.14	-0.15	-0.25	0.24	-0.01	-0.23	0.06	-0.27	-0.33	-0.32	-0.24	-0.21	-0.26	0.45	-0.01	-0.05	-0.16	0.23	-0.17	-0.22	-0.15	-0.10	0.04	-0.19	-0.22	-0.28	0.25		-0.14	-0.11	1.00	-0.08	-0.14
SzegedIndex	0.44	0.42	0.44	0.58	0.13	0.73	0.72	0.73	-0.16	0.88	-0.74	0.10	0.89	0.58	0.34	0.24	0.47	-0.20	-0.09	-0.16	-0.28	0.80	0.91	-0.06	0.96	0.53	0.97	0.02	-0.07	0.62	0.80	0.04	0.53	0.60	0.55	0.41	0.89	0.83	0.39	0.77	0.69	0.93	0.11	0.96	0.91	0.82	0.77	0.70	0.80	0.60	-0.31	-0.16	-0.14		-0.03	-0.16	0.91	0.99
TautomerCount	0.12	0.26	-0.05	-0.09	-0.30	0.04	0.04	0.04	-0.13	-0.09	-0.02	-0.35	-0.10	-0.12	-0.24	0.23	0.22	-0.17	0.74	0.53	0.42	-0.04	0.00	-0.21	-0.04	-0.01	0.03	-0.06	-0.20	0.07	0.13	0.47	-0.07	-0.17	-0.30	-0.42	0.01	0.13	0.05	-0.07	-0.18	-0.06	0.44	0.00	-0.01	-0.13	-0.16	-0.18	0.01	0.18	0.04	-0.14	-0.11	-0.03		-0.10	-0.07	0.02
Tetrahedr.Stereoisom.Count	0.12	0.14	0.44	0.43	0.62	-0.51	-0.51	-0.50	0.89	-0.04	0.15	-0.09	-0.04	-0.48	-0.13	0.03	-0.03	0.86	-0.09	0.02	0.11	-0.03	-0.11	0.26	-0.10	0.14	-0.16	-0.25	0.23	0.00	-0.25	0.07	-0.30	-0.35	-0.35	-0.27	-0.23	-0.27	0.46	-0.01	-0.07	-0.18	0.25	-0.18	-0.24	-0.16	-0.11	0.02	-0.20	-0.22	-0.28	0.24	1.00	-0.16	-0.10		-0.10	-0.16
van der Waals volume	0.39	0.34	0.55	0.67	0.16	0.61	0.60	0.58	-0.09	0.95	-0.70	0.25	0.95	0.53	0.31	0.32	0.53	-0.13	-0.16	-0.19	-0.39	0.71	0.89	-0.12	0.91	0.45	0.88	-0.01	-0.11	0.74	0.84	-0.11	0.38	0.52	0.55	0.47	0.95	0.85	0.41	0.89	0.77	0.94	-0.03	0.93	0.91	0.74	0.68	0.61	0.74	0.69	-0.17	-0.19	-0.08	0.91	-0.07	-0.10		0.91
WienerIndex	0.48	0.47	0.50	0.61	0.06	0.68	0.67	0.68	-0.15	0.88	-0.69	0.07	0.89	0.53	0.26	0.34	0.56	-0.21	-0.05	-0.10	-0.24	0.76	0.91	-0.15	0.94	0.49	0.99	-0.02	-0.16	0.62	0.83	0.09	0.48	0.56	0.50	0.35	0.89	0.85	0.44	0.76	0.67	0.91	0.17	0.95	0.88	0.76	0.69	0.61	0.79	0.68	-0.31	-0.23	-0.14	0.99	0.02	-0.16	0.91	

$p < 0.05$

$p < 0.01$
