# Supplementary material for: Classification of Drugs Based on Properties of Sodium Channel Inhibition: A Comparative Automated Patch-Clamp Study
Source: PLoS One. 2010 Dec 20;5(12):e15568. doi: 10.1371/journal.pone.0015568 (PMC3004914; doi:10.1371/journal.pone.0015568)
Supplement: Results S4 — Classification of 58 chemical descriptors based on their correlations with individual properties of inhibition. (PDF) [file pone.0015568.s007.pdf]

Results S4 – Classification of 58 chemical descriptors based on their correlations with individual properties of inhibition.

Correlations of the 58 chemical descriptors with biophysical properties.
For calculating correlations we used Rev, UD, as well as logarithmically transformed values for IC_{50} , K_r, K_i SD and τ_{off}. Most relevant descriptors, including the ones discussed in the text are colored. Numerical values of descriptors are given in Results S6, correlations are given in Results S5.

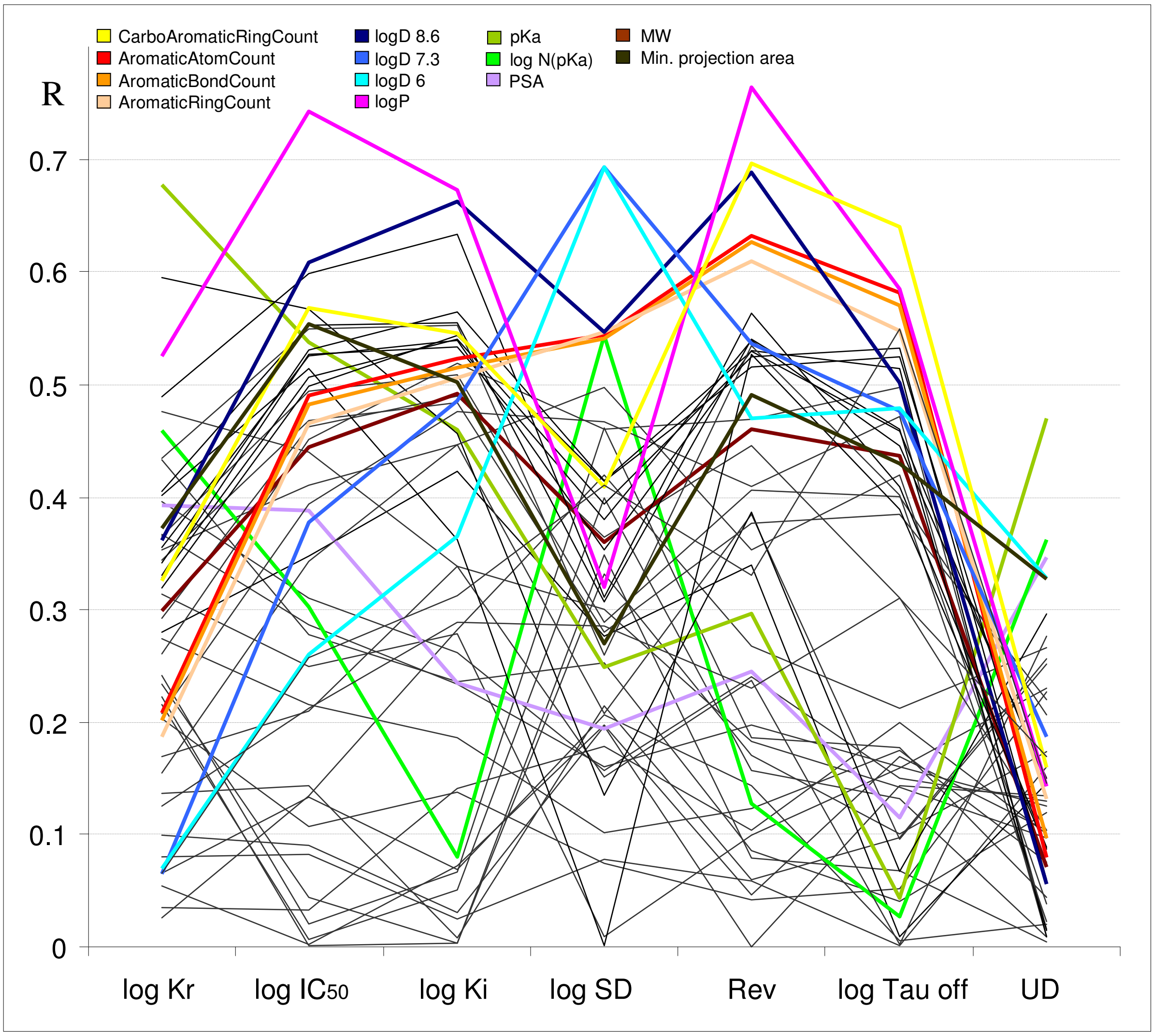

The selection of relevant descriptors was based on the degree of correlation, and on the uniqueness of correlation-patterns. A cluster analysis of descriptors using correlations with individual properties of inhibition as variables confirmed the relative independence of the chosen descriptors.
