# Supplementary material for: Classification of Drugs Based on Properties of Sodium Channel Inhibition: A Comparative Automated Patch-Clamp Study
Source: PLoS One. 2010 Dec 20;5(12):e15568. doi: 10.1371/journal.pone.0015568 (PMC3004914; doi:10.1371/journal.pone.0015568)
Supplement: Results S5 — Correlation coefficients between chemical descriptors and biophysical properties. (PDF) [file pone.0015568.s008.pdf]

Results S5 – Correlation coefficients between chemical descriptors and biophysical properties.

	log IC_{50}	Rev	log τ_{on}	log τ_{off}	UD	log K_r	log $K_{i\text{-}\Delta V1/2}$	log $K_{i\text{-}Kapp}$	log SD
AcceptorCount	-0.03	-0.10	0.16	0.20	-0.10	-0.03	-0.15	-0.15	0.18
AcceptorSiteCount	0.09	0.00	0.06	0.14	-0.13	0.10	-0.02	-0.03	0.20
AliphaticAtomCount	-0.11	0.04	0.06	0.05	0.23	-0.20	0.01	-0.15	-0.07
AliphaticBondCount	-0.25	-0.10	0.19	0.16	0.23	-0.31	-0.10	-0.28	-0.01
AliphaticRingCount	-0.28	-0.09	0.04	-0.04	0.12	-0.43	-0.11	-0.23	-0.25
AromaticAtomCount	-0.49	-0.63	0.61	0.58	-0.08	-0.21	-0.48	-0.51	0.54
AromaticBondCount	-0.48	-0.63	0.59	0.57	-0.10	-0.20	-0.47	-0.50	0.54
AromaticRingCount	-0.47	-0.61	0.55	0.55	-0.13	-0.19	-0.47	-0.49	0.55
AsymmetricAtomCount	0.00	0.17	0.04	-0.10	0.17	-0.22	0.10	-0.01	-0.33
AtomCount	-0.47	-0.38	0.47	0.38	0.22	-0.34	-0.31	-0.49	0.28
BalabanIndex	0.60	0.56	-0.49	-0.41	-0.09	0.49	0.51	0.62	-0.31
BasicpKa	-0.54	-0.30	0.30	0.04	0.47	-0.68	-0.42	-0.50	-0.25
BondCount	-0.49	-0.41	0.49	0.40	0.21	-0.37	-0.34	-0.51	0.29
CarboAromaticRingCount	-0.57	-0.70	0.71	0.64	0.16	-0.32	-0.47	-0.52	0.41
CarboRingCount	-0.51	-0.53	0.50	0.39	0.09	-0.40	-0.25	-0.35	0.00
ChainAtomCount	0.08	0.06	0.00	0.00	0.16	0.08	0.05	0.01	0.08
ChainBondCount	0.00	-0.05	0.14	0.17	0.10	0.05	0.00	-0.08	0.20
ChiralCenterCount	-0.02	0.19	-0.04	-0.18	0.04	-0.24	0.16	0.05	-0.46
DominantTautomerCount	0.44	0.24	-0.18	0.00	-0.28	0.48	0.37	0.37	0.15
DonorCount	0.35	0.34	-0.11	-0.01	-0.15	0.28	0.48	0.43	-0.28
DonorSiteCount	0.41	0.38	-0.22	-0.10	-0.25	0.35	0.46	0.45	-0.21
DreidingEnergy	-0.46	-0.45	0.35	0.31	0.10	-0.29	-0.38	-0.49	0.36
ExactMass	-0.44	-0.46	0.47	0.44	0.07	-0.30	-0.40	-0.49	0.36
FusedRingCount	-0.31	-0.24	-0.01	-0.15	0.13	-0.40	-0.33	-0.27	-0.16
HararyIndex	-0.51	-0.52	0.56	0.53	0.06	-0.33	-0.40	-0.53	0.39
HeteroRingCount	-0.21	-0.18	0.11	0.14	-0.10	-0.17	-0.30	-0.33	0.30
HyperWienerIndex	-0.34	-0.35	0.39	0.42	-0.04	-0.15	-0.28	-0.44	0.50
LargestRingSize	-0.14	0.06	-0.03	-0.10	0.26	-0.14	-0.07	0.00	-0.21
LargestRingSystemSize	-0.29	-0.20	-0.02	-0.16	0.13	-0.37	-0.30	-0.24	-0.16
Length perpendicular to the max area	-0.34	-0.36	0.45	0.30	0.14	-0.15	-0.25	-0.34	0.35
Length perpendicular to the min area	-0.40	-0.39	0.43	0.41	0.06	-0.23	-0.32	-0.45	0.38
log N(pKa)	0.30	0.13	-0.18	-0.03	-0.36	0.46	0.07	0.10	0.54
logD 6	-0.26	-0.47	0.29	0.48	-0.33	0.07	-0.43	-0.35	0.69
logD 7.3	-0.38	-0.54	0.36	0.48	-0.19	-0.06	-0.54	-0.47	0.69
logD 8.6	-0.61	-0.69	0.50	0.50	0.06	-0.36	-0.72	-0.66	0.55
logP	-0.74	-0.76	0.60	0.58	0.14	-0.53	-0.73	-0.67	0.32
Max. projection area	-0.46	-0.42	0.46	0.43	0.20	-0.34	-0.39	-0.51	0.33
Max. projection radius	-0.41	-0.42	0.46	0.44	0.04	-0.23	-0.33	-0.47	0.42
MicrospeciesCount	0.13	0.14	-0.02	0.01	-0.02	0.07	0.09	-0.07	0.21
Min. projection area	-0.55	-0.49	0.55	0.43	0.33	-0.37	-0.41	-0.49	0.27
Min. projection radius	-0.43	-0.39	0.40	0.32	0.39	-0.36	-0.37	-0.41	0.15
MolecularPolarizability	-0.53	-0.53	0.54	0.46	0.14	-0.34	-0.44	-0.56	0.41
PSA 7.3	0.39	0.24	-0.25	-0.11	-0.35	0.39	0.26	0.24	0.19
RandicIndex	-0.50	-0.52	0.56	0.52	0.10	-0.32	-0.40	-0.54	0.41
Refractivity	-0.53	-0.54	0.52	0.45	0.15	-0.33	-0.44	-0.53	0.38
RingAtomCount	-0.53	-0.53	0.54	0.51	-0.01	-0.36	-0.41	-0.52	0.35
RingBondCount	-0.55	-0.54	0.51	0.46	0.01	-0.40	-0.45	-0.54	0.31
RingCount	-0.55	-0.54	0.46	0.40	-0.02	-0.43	-0.44	-0.54	0.26
RingSystemCount	-0.26	-0.33	0.46	0.55	-0.15	-0.07	-0.14	-0.29	0.41
RotatableBondCount	-0.21	-0.23	0.37	0.31	0.17	-0.13	-0.15	-0.29	0.28
SmallestRingSize	0.13	0.27	-0.12	-0.21	0.27	0.03	0.19	0.25	-0.42
SmallestRingSystemSize	-0.22	-0.12	-0.07	-0.17	0.08	-0.27	-0.25	-0.19	-0.10
StereoIsomerCount	-0.04	0.08	-0.02	-0.07	0.00	-0.23	0.11	0.00	-0.35
SzegedIndex	-0.45	-0.47	0.49	0.50	-0.02	-0.26	-0.36	-0.51	0.46
TautomerCount	0.57	0.39	-0.28	-0.07	-0.30	0.59	0.46	0.48	0.13
TetrahedralStereoisomerCount	-0.01	0.16	-0.06	-0.13	0.01	-0.22	0.17	0.04	-0.40
van der Waals volume	-0.51	-0.46	0.53	0.44	0.20	-0.36	-0.39	-0.53	0.33
WienerIndex	-0.39	-0.41	0.45	0.47	-0.01	-0.21	-0.31	-0.47	0.47

$p < 0.05$

$p < 0.01$

$p < 0.001$
