# Supplementary material for: Classification of Drugs Based on Properties of Sodium Channel Inhibition: A Comparative Automated Patch-Clamp Study
Source: PLoS One. 2010 Dec 20;5(12):e15568. doi: 10.1371/journal.pone.0015568 (PMC3004914; doi:10.1371/journal.pone.0015568)
Supplement: Results S6 — Values of selected chemical descriptors for the drugs studied. (PDF) [file pone.0015568.s009.pdf]

Results S6 - Values of selected chemical descriptors for the drugs studied.

	Drugs	Code	MW[a]	pKa[b]	logP[c]	PSA[d]	$logD_{7.3}$[e]	Min. projection area	Aromatic atom count	N(pKa)[f]
ANTIDEPRESSANTS	fluoxetine	**FLX**	309.3	9.8	4.17	25.8	1.74	43.86	12	0.003
	sertraline	**SRT**	306.2	9.85	5.15	16.6	2.68	49.61	12	0.003
	paroxetine	**PRX**	329.4	9.77	3.15	44.3	0.74	53.2	12	0.003
	amitriptyline	**AMI**	277.4	9.76	4.81	4.4	2.39	58.17	12	0.003
	imipramine	**IMI**	280.4	9.2	4.28	7.7	2.39	57.5	12	0.013
	desipramine	**DMI**	266.4	10.02	3.9	19.9	1.29	55.68	12	0.002
	maprotiline	**MPR**	277.4	10.54	4.37	16.6	1.43	49.61	12	0.001
	nisoxetine	**NIS**	271.4	9.8	3.14	35.1	0.71	48.8	12	0.003
	mianserin	**MIA**	264.4	8.12	3.83	7.7	2.90	45.54	12	0.132
	mirtazapine	**MRZ**	265.4	7.8	3.21	20.6	2.53	45.08	12	0.239
	bupropion	**BPR**	239.7	8.22	3.27	33.7	2.30	42.6	6	0.108
	venlafaxine	**VFX**	276.4	8.91	2.74	33.9	1.13	55.77	6	0.024
	nefazodone	**NFZ**	470.0	7.09	4.65	51.6	4.45	75.13	17	0.62
	trazodone	**TRZ**	371.9	7.09	3.13	42.4	2.92	46.72	15	0.62
ANTIPSY.[g]	haloperidol	**HAL**	375.9	8.85	3.66	41.7	2.10	50.1	12	0.028
	chlorpromazine	**CPM**	318.9	9.2	4.54	33.0	2.64	54.73	12	0.013
	chlorprothixene	**CHX**	315.9	9.76	5.07	29.7	2.64	44.52	12	0.003
	clozapine	**CLZ**	326.8	10.1	3.52	33.7	1.01	56.42	12	0.0004
AC[h]	carbamazepine	**CBZ**	236.3	0.31	2.77	46.3	2.77	23.58	12	1
	lamotrigine	**LTG**	256.1	4.67	1.93	90.7	1.93	33.05	12	0.998
	phenytoin	**DPH**	252.3	-5.01	2.15	58.2	2.05	46.84	12	0.94
LA / AAR[i]	bupivacaine	**BPV**	288.4	8.00	3.22	33.5	2.43	60.44	6	0.165
	lidocaine	**LID**	234.3	7.75	1.54	33.5	0.96	46.94	6	0.26
	mexiletine	**MEX**	179.3	9.52	2.46	36.9	0.30	36.55	6	0.006
	flecainide	**FLC**	414.3	9.62	3.19	64.2	0.92	58.51	6	0.005
MISCELLANEOUS	ranolazine	**RAN**	427.5	7.17	1.53	74.3	1.29	56.78	12	0.577
	memantine	**MEM**	179.3	10.7	2.07	27.6	-0.81	42.3	0	0.0004
	riluzole	**RIL**	234.2	4.57	3.4	76.4	3.40	29.52	9	0.999
	diclofenac	**DIC**	296.1	-2.18	4.26	52.2	1.16	43.31	12	0.0005
	ritanserin	**RIT**	477.6	8	5.31	62.4	4.53	66.86	21	0.166
	ambroxol	**AMB**	378.1	9.3	2.65	62.9	0.67	42.95	6	0.01
	silperisone	**SIL**	265.4	10.4	4.86	4.4	1.80	40.43	6	0.001
	tolperisone	**TOL**	245.4	8.78	3.57	21.5	2.08	45.07	6	0.032
	flunarizine	**FLR**	404.5	7.6	6.17	7.7	5.69	61.27	18	0.335
	lifarizine	**LIF**	436.6	8.12	5.89	36.4	5.00	75.97	23	0.128
DROPOUT	mecamylamine	**MEC**	167.3	10.9	2.37	16.6	-0.70	40.90	0	0.0003
	tiapride	**TIA**	328.4	7.64	0.46	85.3	-0.04	55.03	6	0.312
	topiramate	**TOP**	339.4	-0.75	0.13	123.9	0.13	51.25	0	1
	zonisamide	**ZON**	212.2	-1.4	0.11	94.6	0.11	31.21	9	1
	procainamide	**PRC**	235.3	9.04	0.95	59.6	-0.79	36.60	6	0.018
	gabapentine	**GAB**	171.2	9.91	-1.51	67.8	-1.51	36.97	0	0.998
	moclobemide	**MCL**	268.7	6.02	1.45	41.6	1.43	32.61	6	0.95
	nialamide	**NIA**	298.3	3.41	0.39	83.1	0.39	48.35	12	1
	deprenyl	**DPR**	187.3	6.67	2.77	3.2	1.38	32.06	6	0.809

Abbr.: [a] molecular weight; [b] acidic dissociation constant; [c] octanol-water partition coefficient; [d] polar surface area; [e] distribution coefficient at pH = 7.3; [f] N(pKa): the fraction of neutral molecules at pH = 7.3; [g] antipsychotic agents; [h] anticonvulsants; [i] local anesthetics and antiarrhythmic agents.
