# Supplementary material for: Classification of Drugs Based on Properties of Sodium Channel Inhibition: A Comparative Automated Patch-Clamp Study
Source: PLoS One. 2010 Dec 20;5(12):e15568. doi: 10.1371/journal.pone.0015568 (PMC3004914; doi:10.1371/journal.pone.0015568)
Supplement: Results S7 — Geometric and arithmetic mean values of Kr, Ki−ΔV1/2 and Ki−Kapp. (PDF) [file pone.0015568.s010.pdf]

Results S7 –Geometric and arithmetic mean values of K_r, $K_{i\text{-}\Delta V1/2}$ and $K_{i\text{-}Kapp}$.

Drug code	cc [a] µM	K_r geom [b] µM	K_r arith [c] µM	± SEM	$K_{i\text{-}\Delta V1/2}$ geom [b] µM	$K_{i\text{-}\Delta V1/2}$ arith [c] µM	± SEM	$K_{i\text{-}Kapp}$ geom [b] µM	$K_{i\text{-}Kapp}$ arith [c] µM	± SEM
					**A N T I D E P R E S S A N T S**					
**FLX**	**30**	210.5	219.7	± 35.6	4.08	7.09	± 3.42	6.05	8.36	± 3.49
**SRT**	**30**	56.41	57.38	± 6.0	1.47	1.59	± 0.38	2.1	2.43	± 0.66
**PRX**	**30**	24.19	32.1	± 12.2	1.99	8.99	± 5.81	0.73	1.63	± 0.79
**AMI**	**10**	133.9	154.6	± 57.5	1.45	1.97	± 1.12	3.34	5.17	± 3.40
	**30**	124.9	128.5	± 22.5	3.69	3.78	± 0.56	4.92	5.13	± 1.01
	**100**	69.62	72.44	± 14.7	1.65	1.87	± 0.67	2	2.08	± 0.39
**IMI**	**30**	107.2	145.7	± 57.6	3.82	6.22	± 1.97	5.98	7.04	± 1.97
**DMI**	**30**	57.03	59.1	± 8.8	8.06	8.55	± 1.80	4.36	4.53	± 0.73
**MPR**	**10**	89.66	128.2	± 70.4	5.63	6.17	± 1.40	3.17	3.66	± 1.05
**NIS**	**100**	194.2	195.5	± 13.2	19.1	19.5	± 2.12	7.1	7.63	± 1.66
**MIA**	**30**	145.9	154.5	± 29.2	4.5	5.15	± 1.36	4.99	5.87	± 1.82
**MRZ**	**100**	316.8	317.4	± 12.2	5.63	5.65	± 0.32	7.62	7.94	± 1.34
**BPR**	**100**	603.6	733.4	± 270.8	26	29.1	± 7.91	25.5	27.4	± 5.89
**VFX**	**100**	888.7	1001	± 285.7	59.5	75.8	± 26.08	63.4	72.8	± 18.12
**NFZ**	**30**	567.4	563	± 305.5	0.87	1.05	± 0.38	0.92	1.1	± 0.37
**TRZ**	**100**	452	528.7	± 189.1	16.6	20.3	± 6.00	14.27	16.9	± 4.54
					**A N T I P S Y C H O T I C S**					
**HAL**	**30**	113.5	128	± 34.6	13.8	16.6	± 4.34	2.96	3.27	± 0.64
**CPM**	**30**	69.64	85.77	± 26.2	0.29	0.33	± 0.08	0.82	1.03	± 0.33
**CHX**	**30**	128.5	144.1	± 33.9	0.73	1.14	± 0.61	1.74	2.21	± 0.91
**CLZ**	**100**	248.6	254.8	± 34.2	11	13.3	± 4.80	12.38	14.1	± 3.93
					**A N T I C O N V U L S A N T S**					
**CBZ**	**300**	1169	1185	± 122.4	39.9	40.1	± 2.32	26.4	26.9	± 2.98
**LTG**	**300**	2267	2330	± 304.9	31.3	37.3	± 13.48	36.7	40.4	± 9.98
**DPH**	**300**	2536	2954	± 731.2	149.6	172.3	± 51.62	70.8	78.1	± 23.23
					**L O C A L A N E S T H E T I C S / A N T I A R R H Y T H M I C S**					
**BPV**	**100**	618.9	634.9	± 67.2	5.85	6.46	± 1.26	7.79	8.21	± 1.20
**LID**	**300**	3192	3338	± 551.1	89.6	113.7	± 38.33	65.74	70.3	± 13.22
**MEX**	**300**	638.2	662.1	± 109.6	26.8	37.26	± 17.91	17.9	19.2	± 4.13
**FLC**	**300**	64.69	65	± 2.5	7.9	9.72	± 1.47	5.7	6.1	± 0.83
					**M I S C E L L A N E O U S**					
**RAN**	**300**	435.5	586.8	± 223.4	66.8	77.9	± 21.89	10.7	19.7	± 10.15
**MEM**	**100**	242.2	247.7	± 23.9	109.8	119.4	± 22.29	28.52	32.6	± 8.16
**RIL**	**30**	373.6	383.7	± 58.7	2.17	2.18	± 0.17	3.43	3.59	± 0.76
	**100**	359.5	360.3	± 17.6	0.48	0.48	± 0.03	1.63	1.66	± 0.16
**DIC**	**100**	3119	3304	± 566.0	81	101.7	± 40.19	87.8	98.4	± 23.85
**RIT**	**30**	205.7	224.8	± 48.2	7.1	7.69	± 1.57	6.1	6.52	± 1.67
**AMB**	**100**	200.9	201.2	± 7.2	12.2	12.8	± 2.12	17.6	18.6	± 3.43
**SIL**	**100**	132.8	135	± 13.7	14	14.6	± 2.45	6.7	7.08	± 1.22
**TOL**	**100**	391.1	399.4	± 46.7	30.9	43.2	± 18.23	21	24.3	± 7.29
**FLR**	**10**	241	245.8	± 27.6	2	2.35	± 0.75	1.75	2	± 0.57
**LIF**	**1**	55.77	63.15	± 20.1	0.91	0.96	± 0.17	0.5	0.53	± 0.11

Abbr.: [a] concentration of drugs; [b] geometric mean; [c] arithmetic mean.
